# Modeling and Computation of Transboundary Industrial Pollution with Emission Permits Trading by Stochastic Differential Game

**DOI:** 10.1371/journal.pone.0138641

**Published:** 2015-09-24

**Authors:** Shuhua Chang, Xinyu Wang, Zheng Wang

**Affiliations:** 1 Research Center for Mathematics and Economics, Tianjin University of Finance and Economics, Tianjin 300222, China; 2 Institute of Policy and Management, Chinese Academy of Sciences, Beijing 100190, China; Kyushu University, JAPAN

## Abstract

Transboundary industrial pollution requires international actions to control its formation and effects. In this paper, we present a stochastic differential game to model the transboundary industrial pollution problems with emission permits trading. More generally, the process of emission permits price is assumed to be stochastic and to follow a geometric Brownian motion (GBM). We make use of stochastic optimal control theory to derive the system of Hamilton-Jacobi-Bellman (HJB) equations satisfied by the value functions for the cooperative and the noncooperative games, respectively, and then propose a so-called fitted finite volume method to solve it. The efficiency and the usefulness of this method are illustrated by the numerical experiments. The two regions’ cooperative and noncooperative optimal emission paths, which maximize the regions’ discounted streams of the net revenues, together with the value functions, are obtained. Additionally, we can also obtain the threshold conditions for the two regions to decide whether they cooperate or not in different cases. The effects of parameters in the established model on the results have been also examined. All the results demonstrate that the stochastic emission permits prices can motivate the players to make more flexible strategic decisions in the games.

## Introduction

In 1991, the United States and Canada concluded a bargain to deal with the transboundary air pollution, which originated at one location and damaged another region’s air quality after a travelling of the pollution. The transboundary pollution, which is defined as “pollution whose physical origin is situated wholly or in part within the area under the jurisdiction of one state and which has adverse effects in the area under the jurisdiction of another state” in the above United States-Canada agreement, is a particularly serious problem now as it gets the people living in regional borders into facing disproportionate pollution problems. It is the transboundary pollution that originates in one region, but it is able to lead the living organisms in other countries to be disordered or discomfortable by its crossing the border through the pathways of water or air.

The differential game can be regarded as an effective instrument to deal with pollution control problems and to examine the reciprocal actions between the dynamic processes of pollution and participants’ behaviors. Differential games are often used to model and analyze the actions in the case of dynamic systems. There are many players with their own goals in the system and the dynamics of players’ states are modeled by a series of differential equations. In a transboundary pollution control problem, the neighboring countries or regions can be seen as the players and they aim at maximizing the joint or their own net present profits under the cooperative and noncooperative games, respectively. In recent years, many researches have been done on how people make decisions to adapt climate change from the viewpoint of game theory. See, for example, [1–9].

Some researchers have paid their attention to the transboundary industrial pollution problems in recent years. For example, [10] first derived the time consistent solutions in a cooperative differential game and first studied the pollution management in a stochastic differential game framework. In [11], a cooperative stochastic differential game of transboundary industrial pollution is presented, and a payment distribution mechanism is derived to maintain the subgame consistency. Additionally, there are several published studies of transboundary pollution problems from other views, such as renewable resource, clean technologies, harmonization of international and domestic law, abatement cost, R&D spillovers and so on (for instance, [12–15]).

As we all know, most types of pollution are caused by the over emissions of industrial waste. For the purpose of improving global environment, some emission permits trading markets have emerged and are developing prosperously in recent years. However, to our best knowledge, there are very few published articles about differential game of transboundary industrial pollution to take the emission permits trading into consideration. In fact, up to now we have only found the paper [16], in which Li extended Yeung’s model in [11] by taking emission permits trading into account, and the paper [17], in which Bernard et al. examined the impact of the strategic interactions between Russia and China in international carbon emission permits market on the pricing of emission permits by proposing a computable economic model. These two models all regard the emission permits price as a constant or a deterministic function.

Our main work in this paper is that we generalize the emission permits price to follow a geometric Brownian motion (GBM), which commonly depicts the path of underlying assets in financial markets. Market data from the most important emission permits trading systems, such as European Union Emissions Trading Scheme (EU ETS), European Climate Exchange (ECX) and Chicago Climate Exchange (CCX), show that the emission permits price is highly volatile. In the cap-and-trade scheme, the total amount of emission is limited for each emitter, and the difference in the efficiency of using energy and emitting CO_2_ for different emitters makes the emission permits become a scarce resource like a commodity in a capital market. The scarcity of emission permits makes their price affected by the supply and demand. Then, the uncertain relationship between supply and demand for emission permits leads to the volatility in price. Some researchers have studied the emission permits price theoretically and empirically, and most of them believe that there should be a stochastic element in the emission permits prices. See [18–22], for example.

The interaction between the natural environment and the pollutants emitted often effects the accumulation process of pollution stock, hence stochastic elements should appear in the process inevitably. Taking this into account, we present a differential game model to describe transboundary industrial pollution problems, in which both the pollution stock and the emission permits price are all stochastic. By applying the classical tools of stochastic optimal control, we derive a system of Hamilton-Jacobi-Bellman (HJB) partial differential equations satisfied by the value functions from the initial stochastic optimal control problems under the cooperative and the noncooperative games. Note that in a differential game model, the open-loop Nash equilibrium is more tractable than a feedback Nash equilibrium because the controls in open-loop equilibrium only depend on time, however, it is not robust when there is a singular perturbation in the states of the system. Furthermore, the open-loop model only allows the players to choose the strategies at the beginning of games, and the players can not receive or utilize new information during the whole time span (see [23] and [24]). Therefore, here we consider a feedback Nash equilibrium in which controls depend on both time and states, so that the players can adjust their strategies to the new information. That is, before making decisions the players should consider the past choices of the other players at any time.

In many differential game models, usually it is assumed that the game horizon is infinite, which can reduce the complexity of the problem, such that the problem can be solved analytically. In addition, [25] listed some tools for obtaining analytical results. However, in the present paper, we ignore the above simplified assumption (that is, now the time variable *t* is finite), and derive a system of time-dependent HJB equations. Moreover, the volatility exists in both pollution and emission permits price dynamics, such that the system of HJB equations become a degenerate parabolic problem which is different from the common one. In this case, we cannot find out the analytical solution to our model. So, we try to solve it numerically. Some discussions about the numerical algorithms of the HJB equations have been made for the past few years. For example, [26] numerically solved a two-persons zero-sum deterministic differential games governed by a HJB equation, and [27] studied the convergence of monotone *P*_1_ finite element methods for Hamilton-Jacobi-Bellman equations governing optimal control problems. Also, [28] presented an upwind finite-difference method, which is based on an explicit finite-difference scheme and is stable under certain constraints on the step sizes of the discretization, for the numerical approximation of Hamilton-Jacobi-Bellman equations arising from optimal feedback control problems.

In this paper, we propose a so-called fitted finite volume method to solve the HJB equations established by ourselves. The innovation of this method is that it couples a finite volume formulation with a fitted local approximation to the solution. On one hand, we implement the local approximation through solving a sequence of two-point boundary value problems defined on each element. On the other hand, the finite volume method possesses a special feature of the local conservativity of the numerical flux, and is becoming more and more popular. The main advantage of this discrete method is that the system matrix of the resulted discrete equation is an *M*-matrix, which guarantees that the discretization is monotonic and the discrete maximum principle is satisfied. See, for instance, [29] for degenerate parabolic problems, [30] for hyperbolic problems, and [31] for elliptic problems. We hope to make a few theoretical and practical contributions to applying the fitted finite volume method to management problems.

The paper is organized as follows. Section 2 provides the cooperative and the noncooperative game formulations, from which the HJB equations are derived. Then, a so-called fitted finite volume method is proposed for the discretization of the HJB equations in Section 3. In Section 4, some numerical experiments are performed to illustrate the efficiency and usefulness of the numerical method, and the results of economic and managerial meanings are also provided in this section. Finally, concluding remarks are given in Section 5.

## The differential game framework and HJB equations

### The basic differential game framework

For the purpose of illustrating the dynamics of pollution and the interactions among the players in a commitment period, we propose a finite-horizon differential game framework. Also, we assume that the game involves two players (countries or regions), which we do believe can be expanded in the future works.

Let *Q*_*i*_(*t*) (*i* = 1,2) denote the production of region *i* during the period [0,*T*], where *T* is the maturity of the game. This production leads to a quantity of by-products, namely emissions *E*_*i*_(*t*). Region *i*’s net revenue arising from the production can be represented by an increasing concave function *R*_*i*_(*Q*_*i*_(*t*)). Following [16] and [32], we assume that the relationship between the production and emissions is linear, and the production revenue function can be expressed by the following quadratic functional form in terms of emissions:

$$\begin{array}{c} R_{i}\left(E_{i}\left(t\right)\right)=A_{i}E_{i}\left(t\right)-\frac{1}{2}E_{i}^{2}\left(t\right), \end{array}$$ (1)

where *A*_*i*_ is a positive constant. Here we relax the restriction of *E*_*i*_(*t*) ∈ [0,*A*_*i*_] proposed in [16] and [32] for the two reasons: (a) we believe that a negative emission means that the greenhouse gases are removed from the earth’s atmosphere permanently, and an alkali works can realize it; (b) the losses of region *i* caused by an excessive emission (*E*_*i*_ > *A*_*i*_) can be offset by the gains from the emission permits market or from the game.

In an emission permits trading scheme, the initial quota *E*_*i*0_, which is a positive constant, is often allocated by the grandfather principle or auction principle. Then, the trading volume of emission permits of region *i* is given by

$$\begin{array}{c} Y_{i}\left(t\right)=E_{i}\left(t\right)-E_{i0}, \end{array}$$ (2)

where *Y*_*i*_ > 0 means that the region *i* purchases the emission permits from the market, and *Y*_*i*_ < 0 means that the region *i* sells the unused emission permits to others, respectively. Furthermore, we assume that the emission permits price *S*(*t*) is stochastic and follows a geometric Brownian motion (GBM):

$$\begin{array}{c} dS\left(t\right)=\mu_{S}S\left(t\right)dt+\sigma_{S}S\left(t\right)dW_{S}, \end{array}$$ (3)

where *μ*_*S*_ and *σ*_*S*_ are two constants and denote the drift rate and the volatility of emission permits price respectively, and *dW*_*S*_ denotes the increment to standard Brownian process.

It is our main work in this paper to extend the emission permits price from a constant or a deterministic function to a stochastic process. With the Kyoto Protocol entering into force in 2005, emission permits have become a scarce resource for most of regions in the world. The scarcity of emission permits makes their prices affected by the supply and demand. Then, the uncertain relationship between supply and demand for emission permits leads to the volatility in price. Just as mentioned in [18], the emission permits market is of great promise and is becoming more and more liquid, such that it may turn into one of the largest commodities markets in the near future. In a more prosperous market, emission permits prices should be determined by the market, which is similar to the situation in a capital market.

The emissions of several kinds of greenhouse gases have been regarded as permits, and the relationships between supply and demand for different kinds of emission permits should be diverse each other, so the price dynamics for each kind of greenhouse gases should be different. Additionally, the emission permits price dynamics of the same gas in different markets should be also distinct due to the difference in trading form, market participants, and so on.

The GBM is the most widely used model of asset price process in capital markets, and it has been used in the Black-Scholes model, too. To depict the problem from a general viewpoint, we assume in this paper that the emission permits price follows the GBM, which can ensure that the emission permits price is positive. Note that this assumption can be modified when some other specific cases are dealt with.

Consequently, the region *i*’s industrial net revenue involves emission permits trading with a stochastic dynamic price process at time *t* can be represented as

$$\begin{array}{cc} \Pi_{i}\left(E_{i}\left(t\right)\right) & =A_{i}E_{i}\left(t\right)-\frac{1}{2}E_{i}^{2}\left(t\right)-S\left(t\right)\left(E_{i}\left(t\right)-E_{i0}\right)\\ & =\left(A_{i}-S\left(t\right)\right)E_{i}\left(t\right)-\frac{1}{2}E_{i}^{2}\left(t\right)+S\left(t\right)E_{i0}. \end{array}$$ (4)

Moreover, let *P*(*t*) denote the stock of pollution in the environment at time *t*. Then, the dynamic process of pollution stock is governed by the following stochastic differential equation:

$$\begin{array}{c} dP\left(t\right)=\left(E_{1}\left(t\right)+E_{2}\left(t\right)-\theta_{P}P\left(t\right)\right)dt+\sigma_{P}P\left(t\right)dW_{P}, \end{array}$$ (5)

where *E*_1_(*t*) and *E*_2_(*t*) denote the emission levels of regions 1 and 2 respectively, *θ*_*P*_ > 0 represents the exponential decay rate of pollution, and the term *σ*_*P*_ *P*(*t*)*dW*_*P*_ stands for the stochastic disturbance of the pollution. The constant *σ*_*P*_ is a noise parameter and denotes the volatility of pollution stock, and *dW*_*P*_ is the increment to the standard Brownian process. As we have known, the accumulation process of pollution stock is complex. For example, weather fluctuations, nature’s capability to refresh the environment and other natural factors may all contribute to the stochastic dynamic evolution of pollution stock, so it is necessary to add the volatility term into Eq (5). Without loss of generality, we assume that the two Brownian processes *W*_*S*_ and *W*_*P*_ are correlative with a correlation coefficient *ρ* > 0, while *ρ* = 0 means they are independent of each other.

According to [33], we suppose that the pollution damage suffered by region *i* at time *t* is given by *D*_*i*_ *P*(*t*), where *D*_*i*_ is a strictly positive parameter. In addition, we also regard the salvage cost at time *T* for the pollution stock as a linear function $g_{i}({\bar{P}}_{i}-P(T)),$ where *g*_*i*_ > 0 and ${\bar{P}}_{i}>0$.

In particular, we set *A*_2_ = *αA*_1_ and *D*_2_ = *βD*_1_ in the process of solving our problem. By means of [34], it is the parameters *α* and *β* that characterize the differences in the two regions’ capacities in generating revenue from production and in bearing the damages from the stock of pollution or from abatement costs. In addition, these differences can be also resulted from the population difference. How the differences effect the regions’ behaviors will be illustrated in Discussions.

Hence, the current objective of region *i* is to find an optimal plan which maximizes the expected present of the flow of instantaneous net revenue. That is, the objective functional and constraint conditions of region *i* are as follows:

$$\begin{array}{cc} & \max_{E_{i}\left(t\right)}E\{\int_{0}^{T}e^{-rt}[\left(A_{i}-S\left(t\right)\right)E_{i}\left(t\right)-\frac{1}{2}E_{i}^{2}\left(t\right)+S\left(t\right)E_{i0}-D_{i}P\left(t\right)]dt\}-g_{i}\left(P\left(T\right)-{\bar{P}}_{i}\right)e^{-rT},\\ & \text{subject}\;\text{to}\;\{\begin{array}{c} dS\left(t\right)=\mu_{S}S\left(t\right)dt+\sigma_{S}S\left(t\right)dW_{S},S\left(0\right)=S_{0},\\ dP\left(t\right)=\left(E_{1}\left(t\right)+E_{2}\left(t\right)-\theta_{P}P\left(t\right)\right)dt+\sigma_{P}P\left(t\right)dW_{P},P\left(0\right)=P_{0}, \end{array} \end{array}$$

where *r* is the social risk-free discount rate, and *t* = 0 is the initial time.

Next we will take advantage of the stochastic optimal control theory to derive the two regions’ value functions under the cooperative and noncooperative games respectively, by virtue of which we can find out the optimal emission paths, such that the regions’ discounted streams of net revenues are maximized.

### The cooperative game

The game theory can be split into two branches, namely the cooperative game and the noncooperative game. In a cooperative game, the players are restricted by legal agreements to adhere to their promises, and their common goal is to achieve the joint optimum. Under a cooperative framework, the two regions seek the optimal emission path to maximize the joint net revenue. Their joint objective functional and constraint conditions can be written as follows:

$$\begin{array}{c} \max_{E_{C1}\left(t\right),E_{C2}\left(t\right)}E\{\int_{0}^{T}e^{-rt}[\left(A_{1}-S\left(t\right)\right)E_{C1}\left(t\right)+\left(A_{2}-S\left(t\right)\right)E_{C2}\left(t\right)-\frac{E_{C1}^{2}\left(t\right)+E_{C2}^{2}\left(t\right)}{2}\\ +\left(E_{10}+E_{20}\right)S\left(t\right)-\left(D_{1}+D_{2}\right)P\left(t\right)]dt\}-\sum_{i=1,2}g_{i}\left(P\left(T\right)-{\bar{P}}_{i}\right)e^{-rT},\\ \text{subject}\;\text{to}\;\{\begin{array}{c} dS\left(t\right)=\mu_{S}S\left(t\right)dt+\sigma_{S}S\left(t\right)dW_{S},S\left(0\right)=S_{0},\\ dP\left(t\right)=\left(E_{C1}\left(t\right)+E_{C2}\left(t\right)-\theta_{P}P\left(t\right)\right)dt+\sigma_{P}P\left(t\right)dW_{P},P\left(0\right)=P_{0}, \end{array} \end{array}$$ (6)

where *E*_*C*1_(*t*) and *E*_*C*2_(*t*) denote the emission levels of regions 1 and 2 in the cooperative game, respectively.

Assume that the joint value function *V*_*C*_(*P*,*S*,*t*) is a twice continuously differentiable function of *P* and *S*. By applying the dynamic programming approach and It$\hat{\text{o}}$'s lemma to solve the above stochastic optimal control problem, we can obtain the following Hamilton-Jacobi-Bellman equation satisfied by the value function *V*_*C*_(*P*,*S*,*t*):

$$\begin{array}{c} \max_{E_{C1},E_{C2}}\{\frac{\partial V_{C}}{\partial t}+\left(E_{C1}+E_{C2}-\theta_{P}P\right)\frac{\partial V_{C}}{\partial P}+\frac{1}{2}\sigma_{P}^{2}P^{2}\frac{\partial^{2}V_{C}}{\partial P^{2}}+\mu_{S}S\frac{\partial V_{C}}{\partial S}+\frac{1}{2}\sigma_{S}^{2}S^{2}\frac{\partial^{2}V_{C}}{\partial S^{2}}\\ +\rho \sigma_{P}\sigma_{S}PS\frac{\partial^{2}V_{C}}{\partial P\partial S}-rV_{C}+F_{C}\left(P,S,E_{C1},E_{C2},t\right)\}=0, \end{array}$$ (7)

with the terminal condition

$$\begin{array}{c} V_{C}\left(P,S,T\right)=-\sum_{i=1,2}g_{i}\left(P\left(T\right)-{\bar{P}}_{i}\right), \end{array}$$ (8)

where $F_{C}(P,S,E_{C1},E_{C2},t)=(A_{1}-S)E_{C1}+(A_{2}-S)E_{C2}-\frac{E_{C1}^{2}+E_{C2}^{2}}{2}+(E_{10}+E_{20})S-(D_{1}+D_{2})P$

We now present the derivation process of the above HJB equation in the following discussions. First of all, the problem at *t* = *T* needs not to be discussed because it is not a decision problem. In fact, the objective functional (6) becomes $-\sum_{i=1,2}g_{i}(P(T)-{\bar{P}}_{i})$ if *t* = *T*. Therefore, if we choose the emission paths *E*_*C*1_ and *E*_*C*2_ optimally, the value function *V*_*C*_ can be written as the following recursive form (dynamical programming principle):

$$\begin{array}{c} V_{C}\left(P\left(t\right),S\left(t\right),t\right)=\max_{E_{C1}\left(t\right),E_{C2}\left(t\right)}\{\int_{t}^{t+\Delta t}e^{-r(\omega -t)}F_{C}\left(P\left(\omega \right),S\left(\omega \right),E_{C1}\left(\omega \right),E_{C2}\left(\omega \right),\omega \right)d\omega \\ +e^{-r\Delta t}V_{C}\left(P\left(t+\Delta t\right),S\left(t+\Delta t\right),t+\Delta t\right)\}. \end{array}$$ (9)

It follows from moving *V*_*C*_(*P*(*t*),*S*(*t*),*t*) to the right hand side of the above equation and dividing the resulting equation by Δ*t* that

$$\begin{array}{c} 0=\max_{E_{C1}\left(t\right),E_{C2}\left(t\right)}\{\frac{1}{\Delta t}\int_{t}^{t+\Delta t}e^{-r\omega }F_{C}\left(P\left(\omega \right),S\left(\omega \right),E_{C1}\left(\omega \right),E_{C2}\left(\omega \right),\omega \right)d\omega \\ +\frac{e^{-r\Delta t}V_{C}\left(P\left(t+\Delta t\right),S\left(t+\Delta t\right),t+\Delta t\right)-V_{C}\left(P\left(t\right),S\left(t\right),t\right)}{\Delta t}\}. \end{array}$$ (10)

Now, we are in the position to find out what happens if Δ*t* approaches to 0. Obviously, we can see from the Mean Value Theorem that

$$\begin{array}{c} \lim_{\Delta t\to 0}\int_{t}^{t+\Delta t}e^{-r\omega }F_{C}\left(P\left(\omega \right),S\left(\omega \right),E_{C1}\left(\omega \right),E_{C2}\left(\omega \right),\omega \right)d\omega =F_{C}\left(P\left(t\right),S\left(t\right),E_{C1}\left(t\right),E_{C2}\left(t\right),t\right). \end{array}$$

To examine the limit of the second term in the right hand side of Eq (10), we make use of It $\hat{\text{o}}$'s lemma to expand *e* ^−*r*Δ*t*^ *V*_*C*_(*P*(*t*+Δ*t*),*S*(*t*+Δ*t*),*t*+Δ*t*) at Δ*t* = 0 and obtain

$$\begin{array}{c} \begin{array}{cc} & \lim_{\Delta t\to 0}\frac{e^{-r\Delta t}V_{C}\left(P\left(t+\Delta t\right),S\left(t+\Delta t\right),t+\Delta t\right)-V_{C}\left(P\left(t\right),S\left(t\right),t\right)}{\Delta t}\\ = & -rV_{C}+\frac{\partial V_{C}}{\partial t}+\left(E_{C1}\left(t\right)+E_{C2}\left(t\right)-\theta_{P}P\right)\frac{\partial V_{C}}{\partial P}+\frac{1}{2}\sigma_{P}^{2}P^{2}\frac{\partial^{2}V_{C}}{\partial P^{2}}+\mu_{S}S\frac{\partial V_{C}}{\partial S}\\ & +\frac{1}{2}\sigma_{S}^{2}S^{2}\frac{\partial^{2}V_{C}}{\partial S^{2}}+\rho \sigma_{P}\sigma_{S}PS\frac{\partial^{2}V_{C}}{\partial P\partial S}, \end{array} \end{array}$$

in which we assume that *V*_*C*_ is twice continuously differentiable. Then, by means of the above two expressions, we can know that as Δ*t* → 0 Eq (10) becomes Eq (7).

**Remark 1** *In our model, the constraint conditions, the terminal condition and* *F*_*C*_(*P*(*t*), *S*(*t*), *E*_*C*1_(*t*), *E*_*C*2_(*t*), *t*) *are infinitely differentiable functions and bounded for a given domain* Ω = (*P*_min_,*P*_max_) × (*S*_min_,*S*_max_) × (0,*T*). *According to [* 35 *], the dynamical programming principle* (9) *should hold*.

**Remark 2** *However, the above conditions can not ensure that the optimal value function* *V*_*C*_ *is twice continuously differentiable. In the future work, we will try to present a sufficiency theorem to overcome this obstacle*.

### The noncooperative game

A noncooperative game means that each player makes his or her own decisions which may be conflicting with others’ ones to some extent. In our model, if the two regions do not cooperate, they should choose the optimal emission levels to maximize their own net revenues. That is, for region 1:

$$\begin{array}{cc} & \max_{E_{N1}\left(t\right)}E\{\int_{0}^{T}e^{-rt}\left[\left(A_{1}-S\left(t\right)\right)E_{N1}\left(t\right)-\frac{E_{N1}^{2}\left(t\right)}{2}+E_{10}S\left(t\right)-D_{1}P\left(t\right)\right]dt\}-g_{1}\left(P\left(T\right)-{\bar{P}}_{1}\right)e^{-rT},\\ & \text{subject}\;\text{to}\;\{\begin{array}{c} dS\left(t\right)=\mu_{S}S\left(t\right)dt+\sigma_{S}S\left(t\right)dW_{S},S\left(0\right)=S_{0},\\ dP\left(t\right)=\left(E_{N1}\left(t\right)+E_{N2}\left(t\right)-\theta_{P}P\left(t\right)\right)dt+\sigma_{P}P\left(t\right)dW_{P},P\left(0\right)=P_{0}, \end{array} \end{array}$$

and for region 2:

$$\begin{array}{cc} & \max_{E_{N2}\left(t\right)}E\{\int_{0}^{T}e^{-rt}\left[\left(A_{2}-S\left(t\right)\right)E_{N2}\left(t\right)-\frac{E_{N2}^{2}\left(t\right)}{2}+E_{20}S\left(t\right)-D_{2}P\left(t\right)\right]dt\}-g_{2}\left(P\left(T\right)-{\bar{P}}_{2}\right)e^{-rT},\\ & \text{subject}\;\text{to}\;\{\begin{array}{c} dS\left(t\right)=\mu_{S}S\left(t\right)dt+\sigma_{S}S\left(t\right)dW_{S},S\left(0\right)=S_{0},\\ dP\left(t\right)=\left(E_{N1}\left(t\right)+E_{N2}\left(t\right)-\theta_{P}P\left(t\right)\right)dt+\sigma_{P}P\left(t\right)dW_{P},P\left(0\right)=P_{0}, \end{array} \end{array}$$

where *E*_*N*1_(*t*) and *E*_*N*2_(*t*) denote the emission levels of regions 1 and 2 in the noncooperative game, respectively.

Similarly, in the noncooperative game, the value function *V*_*N*1_ and *V*_*N*2_ for two regions 1 and 2 are assumed to be twice continuously differentiable. Through the same discussion as the cooperative case, we can obtain the system of HJB equations satisfied by *V*_*N*1_ and *V*_*N*2_ under the noncooperative game as follows:

$$\left\{ \begin{array}{l} {\text{max}}_{E_{N1}(t)}\{\frac{\partial V_{N1}}{\partial t}+(E_{N1}(t)+E_{N2}(t)-\theta_{P}P)\frac{\partial V_{N1}}{\partial P}+\frac{1}{2}\sigma_{P}^{2}P^{2}\frac{\partial^{2}V_{N1}}{\partial P^{2}}+\mu_{S}S\frac{\partial V_{N1}}{\partial S}+\frac{1}{2}\sigma_{S}^{2}S^{2}\frac{\partial^{2}V_{N1}}{\partial S^{2}}\;+\rho \sigma_{P}\sigma_{S}PS\frac{\partial^{2}V_{N1}}{\partial P\partial S}-rV_{N1}+F_{N1}(P,S,E_{N1}(t),E_{N2}(t),t)\}=0,\\ {\text{max}}_{E_{N2}(t)}\{\frac{\partial V_{N2}}{\partial t}+(E_{N1}(t)+E_{N2}(t)-\theta_{P}P)\frac{\partial V_{N2}}{\partial P}+\frac{1}{2}\sigma_{P}^{2}P^{2}\frac{\partial^{2}V_{N2}}{\partial P^{2}}+\mu_{S}S\frac{\partial V_{N2}}{\partial S}+\frac{1}{2}\sigma_{S}^{2}S^{2}\frac{\partial^{2}V_{N2}}{\partial S^{2}}\;+\rho \sigma_{P}\sigma_{S}PS\frac{\partial^{2}V_{N2}}{\partial P\partial S}-rV_{N2}+F_{N2}(P,S,E_{N1}(t),E_{N2}(t),t)\}=0 \end{array}\right.$$ (11)

with the terminal conditions

$$V_{1}\left(P,S,T\right)=-g_{1}\left(P-{\bar{P}}_{1}\right)\;\text{and}\;V_{2}\left(P,S,T\right)=-g_{2}\left(P-{\bar{P}}_{2}\right),$$

where

$$F_{N1}\left(P,S,E_{N1}\left(t\right),E_{N2}\left(t\right),t\right)=\left(A_{1}-S\left(t\right)\right)E_{N1}\left(t\right)-\frac{E_{N1}^{2}\left(t\right)}{2}+E_{10}S\left(t\right)-D_{1}P\left(t\right),$$

and

$$F_{N2}\left(P,S,E_{N1}\left(t\right),E_{N2}\left(t\right),t\right)=\left(A_{2}-S\left(t\right)\right)E_{N2}\left(t\right)-\frac{E_{N2}^{2}\left(t\right)}{2}+E_{20}S\left(t\right)-D_{2}P\left(t\right).$$

## Numerical methods

In this section, we will present a numerical method to discretize the above HJB equations established by us for the reason that these equations cannot be solved analytically. In fact, here a fitted finite volume method will be employed. Also, it will be shown that the system matrix of the resulting discrete equations is an *M*-matrix, which guarantees that the discretization is monotonic and the discrete maximum principle is satisfied, such that the scheme has a unique solution. Besides, a two-level implicit time-stepping method is used to implement the time-discretization. Since the structure of HJB equations arising from the noncooperative case is similar to the cooperative one, here we only discuss the latter to save the space.

Let us denote the optimal emission paths by $E_{C1}^{*}$ and $E_{C2}^{*}$. From the first-order optimality condition, we know that Eq (7) can be split into the following coupled equations:

$$\begin{array}{c} \frac{\partial V_{C}}{\partial t}+\left(E_{C1}^{*}\left(t\right)+E_{C2}^{*}\left(t\right)-\theta_{P}P\right)\frac{\partial V_{C}}{\partial P}+\frac{1}{2}\sigma_{P}^{2}P^{2}\frac{\partial^{2}V_{C}}{\partial P^{2}}+\mu_{S}S\frac{\partial V_{C}}{\partial S}+\frac{1}{2}\sigma_{S}^{2}S^{2}\frac{\partial^{2}V_{C}}{\partial S^{2}}\\ +\rho \sigma_{P}\sigma_{S}PS\frac{\partial^{2}V_{C}}{\partial P\partial S}-rV_{C}+F_{C}\left(P,S,E_{C1}^{*}\left(t\right),E_{C2}^{*}\left(t\right),t\right)=0, \end{array}$$ (12a)

$$\begin{array}{c} E_{C1}^{*}\left(P,S,t\right)=A_{1}-S+\frac{\partial V_{C}}{\partial P},\;E_{C2}^{*}\left(P,S,t\right)=A_{2}-S+\frac{\partial V_{C}}{\partial P}. \end{array}$$ (12b)

### The fitted finite volume method for spatial discretization

A defined mesh for (*P*_min_,*P*_max_)×(*S*_min_,*S*_max_) is significant in the process of discretization. So, we first divide the intervals *I*_*P*_ and *I*_*S*_ into *N*_*P*_ and *N*_*S*_ sub-intervals, respectively:

$$I_{P_{i}}:=\left(P_{i},P_{i+1}\right),\;\;I_{S_{j}}:=\left(S_{j},S_{j+1}\right),\;\;\;\;i=0,1,\cdots ,N_{P}-1,\;\;j=0,1,\cdots ,N_{S}-1,$$

in which

$$P_{\min}=P_{0}<P_{1}<\cdots <P_{N_{P}}=P_{\max}\;\;\;\;\text{and}\;\;\;\;S_{\min}=S_{0}<S_{1}<\cdots <S_{N_{S}}=S_{\max}.$$

Thus, a mesh on *I*_*P*_ × *I*_*S*_, whose all mesh lines are perpendicular to the axes, is defined. Next we define another partition of *I*_*P*_ × *I*_*S*_ by letting

$$P_{i-\frac{1}{2}}=\frac{P_{i-1}+P_{i}}{2},\;P_{i+\frac{1}{2}}=\frac{P_{i}+P_{i+1}}{2},\;S_{j-\frac{1}{2}}=\frac{S_{j-1}+S_{j}}{2},\;S_{j+\frac{1}{2}}=\frac{S_{j}+S_{j+1}}{2}$$

for any *i* = 1,2,⋯,*N*_*P*_−1 and *j* = 1,2,⋯,*N*_*S*_−1. To keep completeness, we also define $P_{-\frac{1}{2}}=P_{\text{min}},$ $P_{N_{P}+\frac{1}{2}}=P_{\text{max}},$ $S_{-\frac{1}{2}}=S_{\text{min}},$ and $S_{N_{S}+\frac{1}{2}}=S_{\text{max}}$. The step sizes are defined by $h_{P_{i}}=P_{i+\frac{1}{2}}-P_{i-\frac{1}{2}}$ and $h_{S_{j}}=S_{j+\frac{1}{2}}-S_{j-\frac{1}{2}}$ for each *i* = 0,1,⋯,*N*_*P*_ and *j* = 0,1,⋯,*N*_*S*_.

Then, for the purpose of formulating finite volume scheme, we write Eq (12a) in the following divergence form:

$$\begin{array}{c} -\frac{\partial V_{C}}{\partial t}-\nabla \cdot \left(A\nabla V_{C}+\underline{b}V_{C}\right)+cV_{C}=F_{C}, \end{array}$$ (13)

where

$$\begin{array}{cc} A=\left(\begin{array}{cc} a_{11} & a_{12}\\ a_{21} & a_{22} \end{array}\right)=\left(\begin{array}{cc} \frac{1}{2}\sigma_{P}^{2}P^{2} & \frac{1}{2}\rho \sigma_{P}\sigma_{S}PS\\ \frac{1}{2}\rho \sigma_{P}\sigma_{S}PS & \frac{1}{2}\sigma_{S}^{2}S^{2} \end{array}\right), & \\ \underline{b}=\left(\begin{array}{c} b_{1}\\ b_{2} \end{array}\right)=\left(\begin{array}{c} E_{C1}^{*}+E_{C2}^{*}-\theta_{P}P-\sigma_{P}^{2}P-\frac{1}{2}\rho \sigma_{P}\sigma_{S}P\\ \mu_{S}S-\sigma_{S}^{2}S-\frac{1}{2}\rho \sigma_{P}\sigma_{S}S \end{array}\right), & \\ c=r+\mu_{S}+2\frac{\partial^{2}V_{c}}{\partial P^{2}}-\theta_{P}-\sigma_{P}^{2}-\sigma_{S}^{2}-\rho \sigma_{P}\sigma_{S}. & \end{array}$$ (14)

It follows from integrating Eq (13) over ${ℛ}_{i,j}=[S_{i-\frac{1}{2}},S_{i+\frac{1}{2}}]\times [\delta_{j-\frac{1}{2}},\delta_{j+\frac{1}{2}}]$ and applying the mid-point quadrature rule to the resulting equation that

$$\begin{array}{c} -\frac{\partial V_{C_{i,j}}}{\partial t}R_{i,j}-\int_{R_{i,j}}\nabla \cdot \left(A\nabla V_{C}+\underline{b}V_{C}\right)dPdS+c_{i,j}V_{C_{i,j}}R_{i,j}=F_{C_{i,j}}R_{i,j} \end{array}$$ (15)

for *i* = 1,2,⋯,*N*_*P*_−1, *j* = 1,2,⋯,*N*_*S*_−1, where $R_{i,j}=(P_{i+\frac{1}{2}}-P_{i-\frac{1}{2}})\times (S_{j+\frac{1}{2}}-S_{j-\frac{1}{2}})$, *c*_*i*,*j*_ = *c*(*P*_*i*_,*S*_*j*_,*t*), *V*_*C*_*i*,*j*__ = *V*_*C*_(*P*_*i*_,*S*_*j*_,*t*), and $F_{C_{i,j}}=F_{C}(P_{i},S_{j},E^{{*}_{C1}},E_{C2}^{*},t)$.

The approximation of the second term in Eq (15) is the key and difficult point of this numerical method. According to the definition of flux $A\nabla V_{C}+\underline{b}V_{C}$ and integrating by parts, we have

$$\begin{array}{cc} \int_{{ℛ}_{i,j}}\nabla \cdot \left(A\nabla V_{C}+\underline{b}V_{C}\right)dSd\delta = & \int_{\partial {ℛ}_{i,j}}\left(A\nabla V_{C}+\underline{b}V_{C}\right)\cdot lds\\ = & \int_{\left(P_{i+\frac{1}{2}},S_{j-\frac{1}{2}}\right)}^{\left(P_{i+\frac{1}{2}},S_{j+\frac{1}{2}}\right)}(a_{11}\frac{\partial V_{C}}{\partial P}+a_{12}\frac{\partial V_{C}}{\partial S}+b_{1}V_{C})dS\\ - & \int_{\left(P_{i-\frac{1}{2}},S_{j-\frac{1}{2}}\right)}^{\left(P_{i-\frac{1}{2}},S_{j+\frac{1}{2}}\right)}(a_{11}\frac{\partial V_{C}}{\partial P}+a_{12}\frac{\partial V_{C}}{\partial S}+b_{1}V_{C})dS\\ + & \int_{\left(P_{i-\frac{1}{2}},S_{j+\frac{1}{2}}\right)}^{\left(P_{i+\frac{1}{2}},S_{j+\frac{1}{2}}\right)}(a_{21}\frac{\partial V_{C}}{\partial P}+a_{22}\frac{\partial V_{C}}{\partial S}+b_{2}V_{C})dP\\ - & \int_{\left(P_{i+\frac{1}{2}},S_{j-\frac{1}{2}}\right)}^{\left(P_{i-\frac{1}{2}},S_{j-\frac{1}{2}}\right)}(a_{21}\frac{\partial V_{C}}{\partial P}+a_{22}\frac{\partial V_{C}}{\partial S}+b_{2}V_{C})dP, \end{array}$$ (16)

where *l* denotes the unit vector outward-normal to ∂ℛ_*i*,*j*_. We approximate the first integral of Eq (16) by a constant:

$$\int_{\left(P_{i+\frac{1}{2}},S_{j-\frac{1}{2}}\right)}^{\left(P_{i+\frac{1}{2}},S_{j+\frac{1}{2}}\right)}(a_{11}\frac{\partial V_{C}}{\partial P}+a_{12}\frac{\partial V_{C}}{\partial S}+b_{1}V_{C})dS\approx (a_{11}\frac{\partial V_{C}}{\partial P}+a_{12}\frac{\partial V_{C}}{\partial S}+b_{1}V_{C})|{}_{\left(P_{i+\frac{1}{2}},S_{j}\right)}\cdot h_{S_{j}}.$$

Now, we are in the position to derive the approximations to $\left(a_{11}\frac{\partial V_{C}}{\partial P}+a_{12}\frac{\partial V_{C}}{\partial S}+b_{1}V_{C}\right)$ at the mid-point, $\left(P_{i+\frac{1}{2}},S_{j}\right)$, of the interval *I*_*P*_*i*__ for any *i* = 0,1,⋯,*N*_*P*_−1. To begin with, the term $a_{11}\frac{\partial V_{C}}{\partial P}+b_{1}V_{C}$ can be approximated by a constant, which means that its derivative equals zero, that is,

$$\left(\frac{1}{2}\sigma_{P}^{2}P^{2}\frac{\partial V_{c}}{\partial P}+(E_{C_{1}}^{*}+E_{C_{2}}^{*}-\theta_{P}P-\sigma_{P}^{2}P-\frac{1}{2}\rho \sigma_{P}\sigma_{S}P)V_{C}\right)\prime \equiv \left(aP^{2}\frac{\partial V_{C}}{\partial P}+b_{1}^{i+\frac{1}{2},j}V_{C}\right)\prime =0,$$ (17a)

$$V_{C}(P_{i},S_{j})=V_{C_{i,j}},\;V_{C}(P_{i+1},S_{j})=V_{C_{i+1},j},$$ (17b)

where $a=\frac{1}{2}\sigma_{P}^{2}$ and $b_{1}^{i+\frac{1}{2},j}=b_{1}(P_{i+\frac{1}{2}},S_{j})$, *V*_*C*_*i*,*j*__ and *V*_*C*_*i*+1,*j*__ denote the values of *V*_*C*_ at (*P*_*i*_,*S*_*j*_) and (*P*_*i*+1_,*C*_*j*_), respectively. A first-order ordinary differential equation can be obtained by integrating both sides of Eq (17a):

$$aP^{2}\frac{\partial V_{C}}{\partial P}+b_{1}^{i+\frac{1}{2},j}V_{C}=C_{1},$$

where *C*_1_ is an arbitrary constant and can be determined by the boundary conditions Eq (17b) as follows ([29]):

$$C_{1}=b_{1}^{i+\frac{1}{2},j}\frac{V_{C_{i+1,j}}e^{-\frac{\alpha_{i,j}}{P_{i+1}}}-V_{C_{i,j}}e^{-\frac{\alpha_{i,j}}{P_{i}}}}{e^{-\frac{\alpha_{i,j}}{P_{i+1}}}-e^{-\frac{\alpha_{i,j}}{P_{i}}}},$$

where $\alpha_{i,j}=\frac{b_{1}^{i+\frac{1}{2},j}}{a}$. Additionally, the derivative $\frac{\partial V_{C}}{\partial S}$ can be approximated by a forward difference

$$\frac{V_{C_{i,j+1}}-V_{C_{i,j}}}{h_{S_{j}}}.$$

As a result, we have

$$\begin{array}{c} \left(a_{11}\frac{\partial V_{C}}{\partial P}+a_{12}\frac{\partial V_{C}}{\partial S}+b_{1}V_{C}\right){|}_{\left(P_{i+\frac{1}{2}},S_{j}\right)}\cdot h_{S_{j}}\approx (b_{1}^{i+\frac{1}{2},j}\frac{V_{C_{i+1,j}}e^{-\frac{\alpha_{i,j}}{P_{i+1}}}-V_{C_{i,j}}e^{-\frac{\alpha_{i,j}}{P_{i}}}}{e^{-\frac{\alpha_{i,j}}{P_{i+1}}}-e^{-\frac{\alpha_{i,j}}{P_{i}}}}+d_{i,j}\frac{V_{C_{i,j+1}}-V_{C_{i,j}}}{h_{S_{j}}})\cdot h_{S_{j}}, \end{array}$$ (18)

where $d=\frac{1}{2}\rho \sigma_{P}\sigma_{S}PS$ and *d*_*i*,*j*_ = *d*(*P*_*i*_,*S*_*j*_). Applying the similar method to the other three terms of Eq (16), we get following results:

$$\begin{array}{c} \left(a_{11}\frac{\partial V_{C}}{\partial P}+a_{12}\frac{\partial V_{C}}{\partial S}+b_{1}V_{C}\right){|}_{\left(P_{i-\frac{1}{2}},S_{j}\right)}\cdot h_{S_{j}}\approx (b_{1}^{i-\frac{1}{2},j}\frac{V_{C_{i,j}}e^{-\frac{\alpha_{i-1,j}}{P_{i}}}-V_{C_{i-1,j}}e^{-\frac{\alpha_{i-1,j}}{P_{i-1}}}}{e^{-\frac{\alpha_{i-1,j}}{P_{i}}}-e^{-\frac{\alpha_{i-1,j}}{P_{i-1}}}}+d_{i,j}\frac{V_{C_{i,j+1}}-V_{C_{i,j}}}{h_{S_{j}}})\cdot h_{S_{j}}, \end{array}$$ (19)

$$\begin{array}{c} \left(a_{21}\frac{\partial V_{C}}{\partial P}+a_{22}\frac{\partial V_{C}}{\partial S}+b_{2}V_{C}\right){|}_{\left(P_{i},S_{j+\frac{1}{2}}\right)}\cdot h_{P_{i}}\approx S_{j+\frac{1}{2}}({\bar{b}}_{i,j+\frac{1}{2}}\frac{S_{j+1}^{{\bar{\alpha }}_{i,j}}V_{C_{i+1,j}}-S_{j}^{{\bar{\alpha }}_{i,j}}V_{C_{i,j}}}{S_{j+1}^{{\bar{\alpha }}_{i,j}}-S_{j}^{{\bar{\alpha }}_{i,j}}}+{\bar{d}}_{i,j}\frac{V_{C_{i,j+1}}-V_{C_{i,j}}}{h_{P_{i}}})\cdot h_{P_{i}}, \end{array}$$ (20)

and

$$\begin{array}{c} \left(a_{21}\frac{\partial V_{C}}{\partial P}+a_{22}\frac{\partial V_{C}}{\partial S}+b_{2}V_{C}\right){|}_{\left(P_{i},S_{j-\frac{1}{2}}\right)}\cdot h_{P_{i}}\approx S_{j-\frac{1}{2}}({\bar{b}}_{i,j-\frac{1}{2}}\frac{S_{j}^{{\bar{\alpha }}_{i,j-1}}V_{C_{i,j}}-S_{j-1}^{{\bar{\alpha }}_{i,j-1}}V_{C_{i,j-1}}}{S_{j}^{{\bar{\alpha }}_{i,j-1}}-S_{j-1}^{{\bar{\alpha }}_{i,j-1}}}+{\bar{d}}_{i,j}\frac{V_{C_{i,j+1}}-V_{C_{i,j}}}{h_{P_{i}}})\cdot h_{P_{i}}, \end{array}$$ (21)

where ${\bar{\alpha }}_{i,j}=\frac{{\bar{b}}_{i,j+\frac{1}{2}}}{{\bar{a}}_{j}}$, $\bar{a}=\frac{1}{2}\sigma_{S}^{2}$, $\bar{b}=\mu -\sigma_{S}^{2}-\frac{1}{2}\rho \sigma_{P}\sigma_{S}$, and ${\bar{d}}_{i,j}=\frac{1}{2}\rho \sigma_{P}\sigma_{S}P_{i}$. Hence, we obtain the following equations by combining Eqs (15), (16), and (18)-(21) together:

$$\begin{array}{cc} & -\frac{\partial V_{C_{i,j}}}{\partial t}R_{i,j}+e_{i-1,j}^{i,j}V_{C_{i-1,j}}+e_{i,j-1}^{i,j}V_{C_{i,j-1}}+e_{i,j}^{i,j}V_{C_{i,j}}+e_{i,j+1}^{i,j}V_{C_{i,j+1}}+e_{i+1,j}^{i,j}V_{C_{i+1,j}}=F_{C_{i,j}}R_{i,j}, \end{array}$$ (22)

where

$$\begin{array}{c} e_{i-1,j}^{i,j}=-b_{1}^{i-\frac{1}{2},j}\frac{e^{-\frac{\alpha_{i-1,j}}{P_{i-1}}}h_{S_{j}}}{e^{-\frac{\alpha_{i-1,j}}{P_{i}}}-e^{-\frac{\alpha_{i-1,j}}{P_{i-1}}}},\;e_{i,j-1}^{i,j}=-S_{j-\frac{1}{2}}{\bar{b}}_{i,j-\frac{1}{2}}\frac{S_{j-1}^{{\bar{\alpha }}_{i,j-1}}h_{P_{i}}}{S_{j}^{{\bar{\alpha }}_{i,j-1}}-S_{j-1}^{{\bar{\alpha }}_{i,j-1}}}, \end{array}$$ (23)

$$\begin{array}{cc} e_{i,j}^{i,j}= & h_{S_{j}}(\frac{b_{1}^{i+\frac{1}{2},j}e^{-\frac{\alpha_{i,j}}{P_{i}}}}{e^{-\frac{\alpha_{i,j}}{P_{i+1}}}-e^{-\frac{\alpha_{i,j}}{P_{i}}}}+\frac{b_{1}^{i-\frac{1}{2},j}e^{-\frac{\alpha_{i-1,j}}{P_{i}}}}{e^{-\frac{\alpha_{i-1,j}}{P_{i}}}-e^{-\frac{\alpha_{i-1,j}}{P_{i-1}}}}+{\bar{d}}_{i,j})\\ & +h_{P_{i}}(S_{j+\frac{1}{2}}\frac{{\bar{b}}_{i,j+\frac{1}{2}}S_{j}^{{\bar{\alpha }}_{i,j}}}{S_{j+1}^{{\bar{\alpha }}_{i,j}}-S_{j}^{{\bar{\alpha }}_{i,j}}}+S_{j-\frac{1}{2}}\frac{{\bar{b}}_{i,j-\frac{1}{2}}S_{j}^{{\bar{\alpha }}_{i,j-1}}}{S_{j}^{\alpha_{i,j-1}}-S_{j-1}^{{\bar{\alpha }}_{i,j-1}}})+c_{i,j}R_{i,j}, \end{array}$$ (24)

$$\begin{array}{c} e_{i,j+1}^{i,j}=-S_{j+\frac{1}{2}}{\bar{b}}_{i,j+\frac{1}{2}}\frac{S_{j+1}^{{\bar{\alpha }}_{i,j}}h_{P_{i}}}{S_{j+1}^{{\bar{\alpha }}_{i,j}}-S_{j}^{{\bar{\alpha }}_{i,j}}},\;e_{i+1,j}^{i,j}=-b_{1}^{i+\frac{1}{2},j}\frac{e^{-\frac{\alpha_{i,j}}{P_{i+1}}}h_{S_{j}}}{e^{-\frac{\alpha_{i,j}}{P_{i+1}}}-e^{-\frac{\alpha_{i,j}}{P_{i}}}}-h_{S_{j}}{\bar{d}}_{i,j}, \end{array}$$ (25)

for *i* = 1,2,⋯,*N*_*P*_ − 1, *j* = 1,2,⋯,*N*_*S*_ − 1. The other elements $e_{m,n}^{i,j}$ equal zeros when *m* ≠ *i*−1, *i*, *i*+1 and *n* ≠ *j*−1, *j*, *j*+1. We can see that system (22) is an (*N*_*P*_−1)^2^ × (*N*_*S*_−1)^2^ linear system of equations for

$$V_{C}={\left(V_{C_{1,1}},\cdots ,V_{C_{1,N_{S}-1}},V_{C_{2,1}},\cdots ,V_{C_{2,N_{S}-1}},\cdots ,V_{C_{N_{P}-1,1}},V_{C_{N_{P}-1,2}},\cdots ,V_{C_{N_{P}-1,N_{S}-1}}\right)}^{⊤}.$$

Note that *V*_*C*_0,*j*__(*t*),*V*_*C*_*i*,0__(*t*),*V*_*C*_0,*N*_*S*___(*t*), and *V*_*C*_*N*_*P*_,0__(*t*) for *i* = 1,2,⋯,*N*_*P*_ and *j* = 1,2,⋯,*N*_*S*_ equal to the given boundary conditions. Obviously, the coefficient matrix of system (22) is penta-diagonal.

**Remark 3** *In the case of* *P* = 0 or *S* = 0, *the method for the approximation to the flux is invalid since the* Eq (17a) *is degenerate. So, we need to re-consider the problem* (17a) *with an extra degree of freedom in the following form:*

$$\left(\frac{1}{2}\sigma_{P}^{2}P^{2}\frac{\partial V_{C}}{\partial P}+(E_{C1}^{*}+E_{C2}^{*}-\theta_{P}P-\sigma_{P}^{2}P-\frac{1}{2}\rho \sigma_{P}\sigma_{S}P)V_{C}\right)\prime =C_{2}.$$ (26)

*Also, the previous discussions should be changed. To keep things simple, we omit the discussions about this case. For more details, see* [29].

### The implicit difference method for time discretization

Next we embark on the time-discretization of the system (22). To this purpose, we first rewrite Eq (22) as

$$\begin{array}{c} -\frac{\partial V_{C_{i,j}}}{\partial t}R_{i,j}+D_{i,j}V_{C}=F_{C_{i,j}}R_{i,j}, \end{array}$$ (27)

where

$$D_{i,j}=\left(0,\cdots ,0,e_{i-1,j}^{i,j},0,\cdots ,0,e_{i,j-1}^{i,j},e_{i,j}^{i,j},e_{i,j+1}^{i,j},0,\cdots ,0,e_{i+1,j}^{i,j},0,\cdots ,0\right)$$

for *i* = 1,2,⋯,*N*_*P*_ − 1 and *j* = 1,2,⋯,*N*_*S*_ − 1. We select *M* − 1 points numbered from *t*_1_ to *t*_*M* − 1_ between 0 and *T*, and let *T* = *t*_0_, *t*_*M*_ = 0 to form a partition of time *T* = *t*_0_ > *t*_1_ > ⋯ > *t*_*M*_ = 0. Then, the full discrete form of Eq (27) can be obtained by applying the two-level implicit time-stepping method with a splitting parameter $\theta \in [\frac{1}{2},1]$ to it:

$$\begin{array}{c} \begin{array}{cc} & \left(\theta D\left(P,S,E_{C1}^{*}\left(t^{m+1}\right),E_{C2}^{*}\left(t^{m+1}\right),t^{m+1}\right)+G^{m}\right)V_{C}^{m+1}\\ = & \theta F_{C}\left(P,S,E_{C1}^{*}\left(t^{m+1}\right),E_{C2}^{*}\left(t^{m+1}\right),t^{m+1}\right)+\left(1-\theta \right)F_{C}\left(P,S,E_{C1}^{*}\left(t^{m}\right),E_{C2}^{*}\left(t^{m}\right),t^{m}\right)\\ & +\left(G^{m}-\left(1-\theta \right)D\left(P,S,E_{C1}^{*}\left(t^{m}\right),E_{C2}^{*}\left(t^{m}\right),t^{m}\right)\right)V_{C}^{m}, \end{array} \end{array}$$ (28)

where

$$\begin{array}{c} V_{C}^{m}={\left(V_{C_{1,1}}^{m},\cdots ,V_{C_{1,N_{S}-1}}^{m},V_{C_{2,1}}^{m},\cdots ,V_{C_{2,N_{S}-1}}^{m},\cdots ,V_{C_{N_{P}-1,1}}^{m},\cdots ,V_{C_{N_{P}-1,N_{S}-1}}^{m}\right)}^{⊤},\\ G^{m}=\text{diag}\;{\left(-R_{1,1}/\Delta t_{m},\cdots ,-R_{N_{P}-1,N_{S}-1}/\Delta t_{m}\right)}^{⊤}, \end{array}$$ (29)

for *m* = 0,1,⋯,*M* − 1. Note that Δ*t*_*m*_ = *t*_*m*+1_ − *t*_*m*_ < 0, and $V_{C}^{m}$ denotes the approximation of *V*_*C*_ at *t* = *t*_*m*_. Particularly, when we set $\theta =\frac{1}{2}$, the scheme (28) becomes the famous Crank-Nicolson scheme and is second-order accuracy; when we set *θ* = 1, the scheme (28) becomes the backward Euler scheme and is first-order accuracy.

The following theorem declares that the system matrix of system (28) is an *M*-matrix.

**Theorem 1** *For any given* *m* = 1,2,⋯,*M* − 1, if ∣Δ*t*_*m*_∣ *is sufficiently small and* *c* ≥ 0, *then the system matrix of* Eq (28) *is an M-matrix*.

**Proof.** First, we note that $e_{m,n}^{i,j}\leq 0$ for all *m* ≠ *i*, *n* ≠ *j*, since

$$\begin{array}{c} \frac{b_{1}^{i+\frac{1}{2},j}}{e^{-\frac{\alpha_{i,j}}{P_{i+1}}}-e^{-\frac{\alpha_{i,j}}{P_{i}}}}>0,\;\frac{{\bar{b}}_{i+\frac{1}{2},j}}{S_{i+1}^{{\bar{\alpha }}_{i,j}}-S_{i}^{{\bar{\alpha }}_{i,j}}}>0 \end{array}$$ (30)

for any *i* and *j*, and for any *α* = *b*_1_/*a* and any $\bar{\alpha }=\bar{b}/\bar{a}$. This is because the function $e^{-\frac{\alpha }{P}}$ is increasing when *b*_1_ > 0 and decreasing when *b*_1_ < 0, and the function $S^{\bar{\alpha }}$ is increasing when $\bar{b}>0$ and decreasing when $\bar{b}<0$. Moreover, Eq (30) also holds when $b_{1}^{i+\frac{1}{2},j}\to 0$, ${\bar{b}}_{i,j+\frac{1}{2}}\to 0$. Furthermore, from Eq (23) to Eq (25) we know that when *c*_*i*,*j*_ ≥ 0, for all *i* = 1,⋯,*N*_*P*_−1, *j* = 1,⋯,*N*_*S*_−1, there holds

$$\begin{array}{cc} {\left(e_{i,j}^{i,j}\right)}^{m+1} & \geq |{\left(e_{i-1,j}^{i,j}\right)}^{m+1}\left|+\right|{\left(e_{i,j-1}^{i,j}\right)}^{m+1}\left|+\right|{\left(e_{i,j+1}^{i,j}\right)}^{m+1}\left|+\right|{\left(e_{i+1,j}^{i,j}\right)}^{m+1}|+c_{i,j}^{m+1}R_{i,j}\\ & =\sum_{p=1}^{N_{S}-1}\sum_{q=1}^{N_{\delta }-1}\left|{\left(e_{p,q}^{i,j}\right)}^{m+1}\right|+c_{i,j}^{m+1}R_{i,j}. \end{array}$$

Therefore, $D(P,S,E_{C1}^{*}(t^{m+1}),E_{C2}^{*}(t^{m+1}),t^{m+1})$ is a diagonally dominant with respect to its columns. Hence, from the above analysis, we see that for all admissible *i*, *j*, $D(P,S,E_{C1}^{*}(t^{m+1}),\backslash E_{C2}^{*}(t^{m+1}),t^{m+1})$ is a diagonally dominant matrix with positive diagonal elements and non-positive off-diagonal elements. This implies that $D(P,S,E_{C1}^{*}(t^{m+1}),E_{C2}^{*}(t^{m+1}),t^{m+1})$ is an *M*-matrix.

Second, *G* ^*m*^ of the system matrix (28) is a diagonal matrix with positive diagonal entries. In fact, when ∣Δ*t*_*m*_∣ is sufficiently small, we have

$$\theta c_{i,j}R_{i,j}+\frac{R_{i,j}}{-\Delta t_{m}}>0,$$

which demonstrates that $\theta D(P,S,E_{C1}^{m+1},E_{C2}^{m+1},t^{m+1})+G^{m}$ is an *M*-matrix.

### Decoupling of the system

In the above discussion, we have assumed that the control variables $E_{C1}^{*}$ and $E_{C2}^{*}$ are known. However, we can see from Eq (28) that $E_{C1}^{*}$ and $E_{C2}^{*}$ are coupled with *V*_*C*_ when *θ* ≠ 0. To deal with this dilemma, we replace $E_{C1}^{*}(t^{m+1})$ and $E_{C2}^{*}(t^{m+1})$ by $E_{C1}^{*}(t^{m})$ and $E_{C2}^{*}(t^{m})$, respectively. This method is proposed by [36], and should be reasonable because the control variables are just replaced by their values in the previous time step. The error is small if Δ*t*_*m*_ is sufficiently small. The resulting system corresponding to Eq (28) is as follows:

$$\begin{array}{c} \begin{array}{cc} & \left(\theta D\left(P,S,E_{C1}^{*}\left(t^{m}\right),E_{C2}^{*}\left(t^{m}\right),t^{m+1}\right)+G^{m}\right)V_{C}^{m+1}\\ = & \theta F\left(P,S,E_{C1}^{*}\left(t^{m}\right),E_{C2}^{*}\left(t^{m}\right),t^{m+1}\right)+\left(1-\theta \right)F\left(P,S,E_{C1}^{*}\left(t^{m}\right),E_{C2}^{*}\left(t^{m}\right),t^{m}\right)\\ & +\left(G^{m}-\left(1-\theta \right)D\left(P,S,E_{C1}^{*}\left(t^{m}\right),E_{C2}^{*}\left(t^{m}\right),t^{m}\right)\right)V_{C}^{m}. \end{array} \end{array}$$ (31)

## Numerical results

Up to now, we have been able to show the results of our differential game model numerically. We use the following parameter values to solve the HJB equations under the cooperative and noncooperative games, respectively, and utilize the results as a benchmark case in the following discussions. Parameters: *T* = 10, *A*_1_ = 5, *α* = 0.95, *E*_10_ = 5, *E*_20_ = 6, *θ* = 0.06, *P*_min_ = 100, *P*_max_ = 1000, *S*_min_ = 0, *S*_max_ = 2, *σ*_*P*_ = 0.3, *σ*_*S*_ = 0.3, *μ*_*S*_ = 0.2, *ρ* = 0.5, *D*_1_ = 0.1, *β* = 1.2, *r* = 0.08, *g*_1_ = 3, *g*_2_ = 2, ${\bar{P}}_{1}=1100$, ${\bar{P}}_{2}=1200$.

### The efficiency of the numerical method

**Fig 1:**
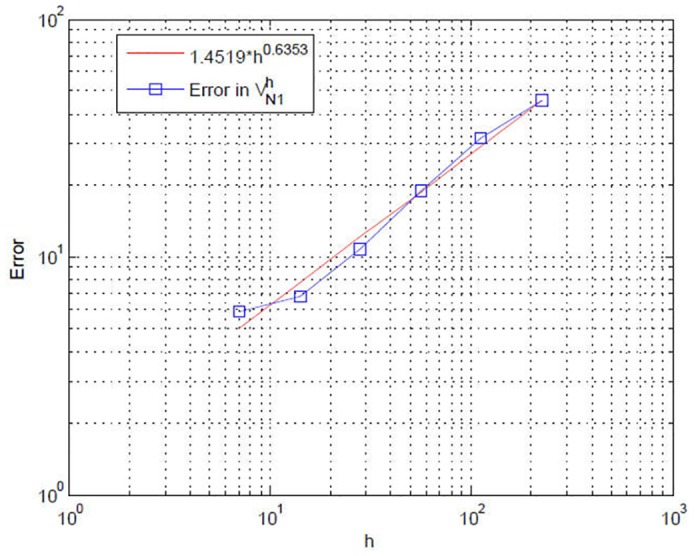
Computed errors in the *L* ^∞^-norm at *t* = 0.

First of all, we consider the convergence rate of our discretization method to show its accuracy and efficiency. Owing to the limitation of space, we only test region 1’s value function *V*_*N*1_ under the noncooperative game. Additionally, since the closed-form solution of the HJB equation cannot be found, we regard the solution of the *N*_*P*_ = 256 = *N*_*S*_ and *M* = 256 mesh in both space and time, respectively, as the “exact” solution *V*_*N*1_. We compute the errors in the discrete *L* ^∞^-norm at the computational final time step *t* = 0 on a sequence of meshes with *N*_*P*_ = *N*_*S*_ = *M* = 2^*n*^ for a positive integer *n* from *n* = 2 to a maximum *n* = 7. The discrete *L* ^∞^-norm is defined as:

$$∥V_{N1}^{h}\left(P,S,0\right)-V_{N1}{\left(P,S,0\right)∥}_{\infty }=\max_{1<i<N_{P},1<j<N_{S}}\left|V_{N1}^{h}\left(P_{i},S_{j},0\right)-V_{N1}\left(P_{i},S_{j},0\right)\right|,$$

where $V_{N1}^{h}$ denotes the numerical solution. The log-log plots of the computed maximum errors, along with the linear fitting, are depicted in Fig 1. From the figure we see that the rate of convergence of $V_{N1}^{h}$ in the discrete *L* ^∞^ norm is of the order 𝒪(*h* ^0.6353^), where $h=\underset{1<i<N_{P},1<j<N_{S}}{\text{max}}(h_{P_{i}},h_{S_{j}})$. Note that this result is reasonable because of the coupling in the HJB equations. Moreover, it numerically demonstrates that our numerical methods for the HJB equations governing the differential game in transboundary industrial pollution is useful and efficient. Some theoretical analysis about convergence rates should be discussed in the future works.

### The solution of the model

In this and next parts, we will illustrate the results by presenting some tables and figures. In Tables 1–4, the values of value functions, emission levels and trading volumes about pollution stock, and permit prices at time *t* = 0 and *t* = 5 are presented. Tables 1 and 2 are for the noncooperative game, and Tables 3 and 4 for the cooperative game, respectively. Note that the trading volumes *Y* for each table are computed by using the Eq (2).

**Table 1:** The noncooperative game at *t* = 0.

pollution stock	permits price	value functions		emission levels		trading volumes	
*P*	*S*	*V*_*N*1_	*V*_*N*2_	*E*_*N*1_	*E*_*N*2_	*Y*_*N*1_	*Y*_*N*2_
325	0.5	1080	745	3.211	2.909	-1.788	-3.091
	1	1093	760	2.729	2.427	-2.271	-3.573
	1.5	1102	769	2.237	1.937	-2.763	-4.063
550	0.5	777	508	3.108	3.009	-1.892	-2.991
	1	792	524	2.599	2.498	-2.401	-3.502
	1.5	800	533	2.095	1.994	-2.905	-4.006
775	0.5	457	309	3.059	3.246	-1.941	-2.754
	1	467	320	2.528	2.710	-2.472	-3.289
	1.5	473	326	2.009	2.190	-2.990	-3.810

**Table 2:** The noncooperative game at *t* = 5.

pollution stock	permits price	value functions		emission levels		trading volumes	
*P*	*S*	*V*_*N*1_	*V*_*N*2_	*E*_*N*1_	*E*_*N*2_	*Y*_*N*1_	*Y*_*N*2_
325	0.5	1612	1159	2.641	2.582	-2.359	-3.418
	1	1625	1173	2.151	2.093	-2.848	-3.907
	1.5	1635	1184	1.657	1.600	-3.343	-4.400
550	0.5	1181	841	2.513	2.608	-2.487	-3.392
	1	1194	856	2.005	2.098	-2.995	-3.902
	1.5	1203	866	1.501	1.594	-3.499	-4.407
775	0.5	713	538	2.327	2.709	-2.673	-3.291
	1	723	548	1.798	2.177	-3.202	-3.823
	105	729	556	1.279	1.656	-3.721	-4.344

**Table 3:** The cooperative game at *t* = 0.

pollution stock	permits price	value functions	emission levels		trading volumes	
*P*	*S*	*V*_*C*_	*E*_*C*1_	*E*_*C*2_	*Y*_*C*1_	*Y*_*C*2_
325	0.5	1835	2.149	1.649	-2.851	-4.351
	1	1865	1.684	1.183	-3.316	-4.816
	1.5	1882	1.200	0.700	-3.799	-5.299
550	0.5	1299	2.117	1.616	-2.883	-4.383
	1	1329	1.596	1.096	-3.403	-4.904
	1.5	1347	1.086	0.586	-3.914	-5.414
775	0.5	777	2.276	1.776	-2.724	-4.224
	1	797	1.709	1.290	-3.290	-4.790
	1.5	810	1.171	0.671	-3.829	-5.329

**Table 4:** The cooperative game at *t* = 5.

pollution stock	permits price	value functions	emission levels		trading volumes	
*P*	*S*	*V*_*C*_	*E*_*C*1_	*E*_*C*2_	*Y*_*C*1_	*Y*_*C*2_
325	0.5	2788	1.243	0.742	-3.757	-5.257
	1	2815	0.764	0.264	-4.236	-5.736
	1.5	2833	0.277	-0.224	-4.724	-6.224
550	0.5	2041	1.115	0.615	-3.885	-5.385
	1	2067	0.599	0.099	-4.401	-5.902
	1.5	2086	0.089	-0.411	-4.911	-6.411
775	0.5	1265	0.997	0.497	-4.003	-5.503
	1	1284	0.439	-0.060	-4.560	-6.060
	1.5	1297	-0.099	-0.599	-5.099	-6.599

To begin with, we can see from each table that a higher permit price results in a more revenue, a lower emission level as well as a larger selling volumes of emission permits for regions 1 and 2 under the noncooperative and the cooperative framework, respectively. In this example, the initial quotas *E*_10_ and *E*_20_ are both set to be very large, and the emission levels do not exceed them, so the two regions can sell their unused emission permits and the net revenues *V*_*Ci*_ and *V*_*Ni*_ will increase with the increasing permits price *S*. To illustrate the problem entirely, we will examine the effects of the initial quotas on the results in the next part. Besides, the first-order conditions of Eqs (7) and (11) show that the optimal emission levels of the two regions can be expressed as

$$\begin{array}{c} E_{Ci}^{*}=A_{i}-S+\frac{\partial V_{Ci}}{\partial P}\;\text{and}\;E_{Ni}^{*}=A_{i}-S+\frac{\partial V_{Ni}}{\partial P} \end{array}$$ (32)

for *i* = 1,2 under the cooperative and noncooperative games, respectively. From the above equations, we can clearly see that the emission levels should decrease monotonically with increasing the permit prices. This implies that the existence of emission permits trading scheme does influence the players’ decisions in the games.

Then, through comparing the results at the different time points, we can know that the value functions are smaller at the initial time point *t* = 0 than at the middle time point *t* = 5 for both the cooperative and the noncooperative games. This demonstrates that the evolution of net revenues is a general accumulated process. In addition, the higher emission levels at early stage can be also seen as an initial investment, which is necessary for both players’ stable developments in the games.

Moreover, there is a complex relationship between the emission level and the pollution stock. In the noncooperative game, the emission level is a decreasing function about the pollution stock for region 1, while it is a increasing function for region 2. This is mainly caused by the differences in abilities between the two regions, which are characterized by *α* and *β*. The parameters *α* < 1 and *β* > 1 mean that region 1 has an advantage over region 2. The advantaged region 1 can reduce the scale of production and lower the emission level when facing to a high concentration of pollution stock, while the disadvantaged region 2 has to enlarge the scale of production to make up the losses caused by the pollution stock, which leads to the vicious spiral. In the cooperative game, the relationship between the emission level and the pollution stock can be described by *U*-shaped curve at the initial time. This is the result of the interaction between the two regions. While at the latter time, both the regions choose to abate the emissions for a high concentration of pollution stock, which is like region 1’s action in the noncooperative game and is a rational response.

We can also know from the tables that the net revenues are undoubtedly decreasing functions of the pollution stock *P*, which is a common view in the most published literature, such as [11] and [16], and so on.

Note that the topic on how to distribute the joint net revenues to each player in the cooperative game has been paid more attentions. One reasonable distribution mechanism is to share the joint net revenues in accordance with the proportion of noncooperative payoffs. This can be expressed mathematically as:

$$V_{Ci}=\frac{V_{Ni}}{V_{N1}+V_{N2}}V_{C}$$ (33)

for *i* = 1,2, where *V*_*Ci*_ denotes region *i*’s value function in the cooperative game. The two regions will be cooperative when *V*_*C*_ > *V*_*N*1_+*V*_*N*2_, and the revenue *V*_*Ci*_ in the cooperative game should be higher than the revenue *V*_*Ni*_ in the noncooperative game.

Coincidentally, the joint net revenue in the cooperative game is always higher than the sum of each net revenue in the noncooperative game in the benchmark case, which means that the players would like to cooperate during the entire game horizon. However, we believe that the players have the potential not to cooperate in the game for the following two reasons.

On the one hand, due to the randomness in emission permits prices, which results from all permits market participants’ behaviors, the players cannot have clear understanding about their optimal net revenues in the process of game. Therefore, it is not only the emission levels but also the decisions on whether to cooperate need to be adjusted based on the states to get the maximum benefits for the two players. On the other hand, there should be some differences between the two regions in practical capabilities to generate revenue from production and to bear the damages from the stock of pollution or from the abatement costs, which is characterized by parameters *α* and *β*, and then the advantaged one may prefer to suspend the cooperation when there exists a free-riding.

In [16], the permits price is a constant, and the cooperation is always a better decision no matter how *α* and *β* vary. In fact, the same results as those in [16] can be obtained when we solve a simple version of our differential game model, in which the permits price is not stochastic, by using our numerical method. However, we will show in the next section that the noncooperation can be also a better decision for the two regions when the permits price is stochastic, and the differences between them, characterized by *α* and *β*, become bigger and bigger. Thus, we have the reason to believe that our stochastic emission permits price can be a better tool to model the emission permits trading part of the differential game, as it can motivate the players to make more flexible decisions such as the noncooperation in the game. Some detailed discussions will be presented in the following section to show the effects of some parameters.

### Discussions

In this subsection, we will examine the effects of parameters on the results. The parameters are divided into three groups: (a) the differences between the two players characterized by *α* and *β*; (b) the emission permits price parameters *μ*_*S*_ and *σ*_*S*_; and (c) the initial quotas of the two regions *E*_10_ and *E*_20_. Besides of the value functions and optimal emission pathes in the noncooperative and the cooperative cases, we also focus on the threshold states, in which the two players should change their decisions from the cooperation to the noncooperation, or vice versa in different cases. Some results will be showed in the following figures, in which *t* = 0 and *P* = 550.

#### the effects of parameters *α* and *β*

As mentioned above, the parameters *α* and *β* represent the differences in abilities between the two regions. We believe that these differences should influence their optimal decisions.

**Fig 2:**
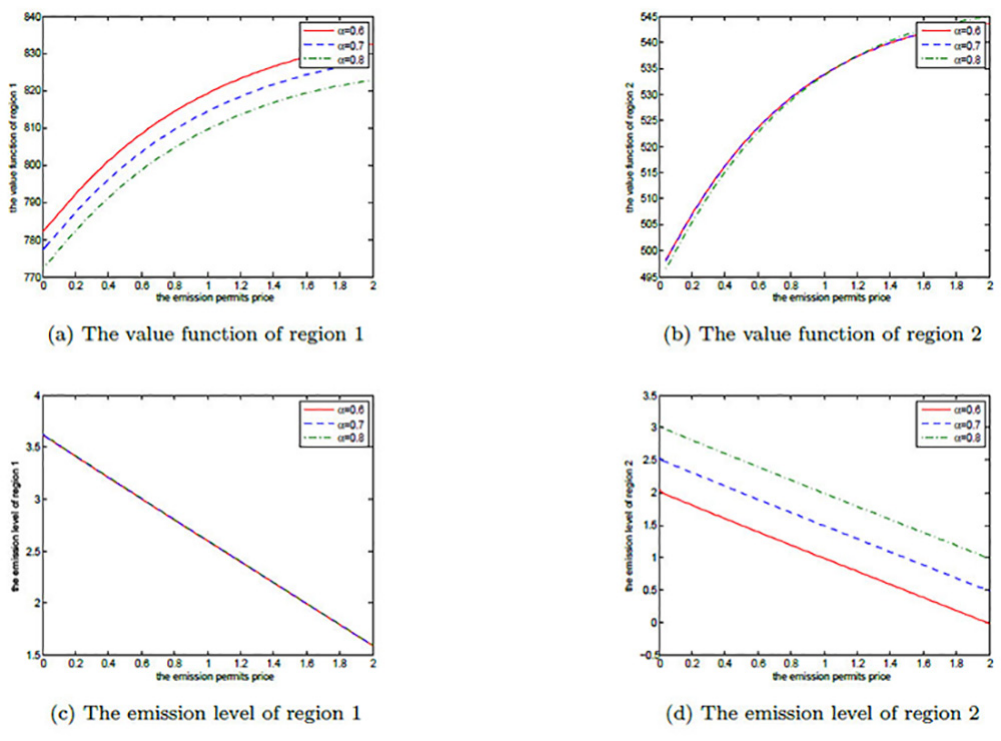
The effects of *α* on the noncooperative game.

**Fig 3:**
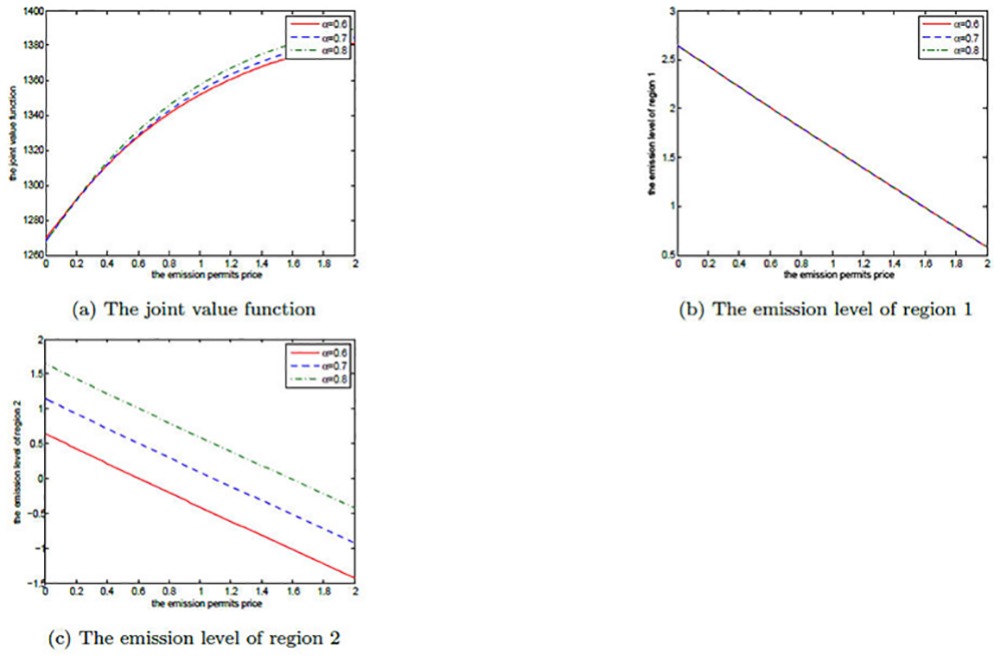
The effects of *α* on the cooperative game.

We first illustrate the effects of *α* on the noncooperative and the cooperative games by Figs 2 and 3, respectively. It is set to be 0.6, 0.7 and 0.8 in each figure. In our model, the parameters *A*_1_ and *A*_2_ measure the increased revenues by adding one unit of emission, and *α* < 1 means that region 1 is more productive than region 2.

In the noncooperative game, increasing *α* implies that region 2’s ability in production is enhanced, and then it increases the emission level to receive more production revenues. At the same time, more pollution stocks are also produced, which leads to more pollution damages. So, region 2’s net revenue is not sensitive to parameter *α*. For region 1, though there is no change in its optimal emission path, it has also suffered from the more pollution damages caused by region 2’s more emissions. Therefore, region 1’s net revenue decreases with the increasing *α*, which is illustrated in Fig 2(a).

In the cooperative game, the two regions stand together to make their net revenues maximum, and region 2 should make use of its enhanced productive ability to improve the joint value function. However, these added net revenues are not so many, as they should be neutralized by more pollution stocks. Similar to the noncooperative case, region 1’s optimal emission path does not change, either. These are presented in Fig 3.

**Fig 4:**
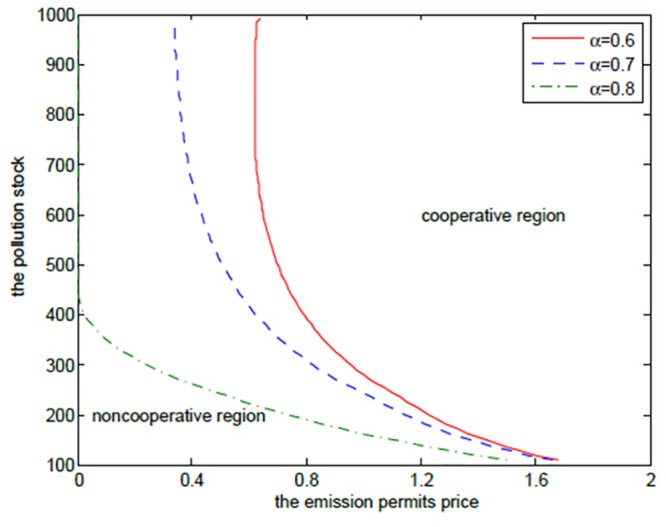
The effects of *α* on the optimal decision boundary.

Although the increasing *α* results in more joint net revenues, it is not always true for any pollution stock *P* in the case that the joint value function *V*_*C*_ is larger than the sum of the net revenues *V*_*N*1_ and *V*_*N*2_ in the noncooperative game. Fig 4 shows the boundaries at which the two players should change their decisions from the cooperation to the noncooperation for different *α* at time *t* = 0. Similar to the optimal exercise boundaries in American options, the curve, which can be called “optimal decision boundary”, divides the domain Ω = (*P*_min_,*P*_max_) × (*S*_min_,*S*_max_) into the cooperative region and the noncooperative region. In the cooperative region, the optimal cooperative net revenue is always higher than the sum of the noncooperative net revenues, and in the noncooperative region, the sum of the noncooperative net revenues is larger than the cooperative one, and on the optimal decision boundary, they are the same.

Fig 4 demonstrates that the two regions should cooperate when the state pollution stock *P* and the emission permits price *S* are larger, and should not cooperate when the states pollution stock *P* and the emission permits price *S* are smaller. This can be illustrated from the following two aspects. On the one hand, for the given higher pollution stock and the emission permits price, the players hope to stand together to lower their emission levels, which is a reasonable action. This can be demonstrated by comparing the noncooperative emission levels with the cooperative ones. We can see from Tables 1–4 that the emission levels in the cooperative game are always lower than the ones in the noncooperative game. On the other hand, the players should try to seek their own optimal net revenues in the case that the emission permits price is at such a lower level that the added revenues in the emission market are not enough to make up the reduced productive revenues resulting from the lower emission level in the cooperative game. Besides, the small amount of pollution stock cannot make the two regions recognize the necessity and urgency of emission reduction. This is the feature of the noncooperative region in Fig 4.

It is clear that the cooperative region expands with *α* increasing. From the above analysis, we can know that the closer *α* approximates to 1, the less the difference between the two regions is in the productive ability, and the greater the willingness for the advantaged region 1 to cooperate with region 2 is.

**Fig 5:**
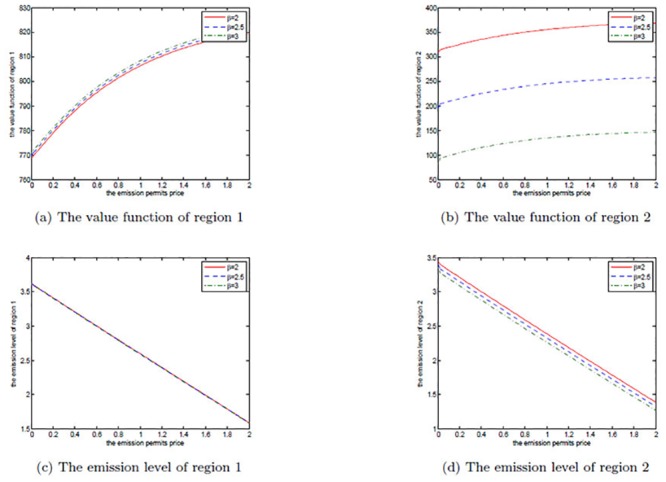
The effects of *β* on the noncooperative game.

**Fig 6:**
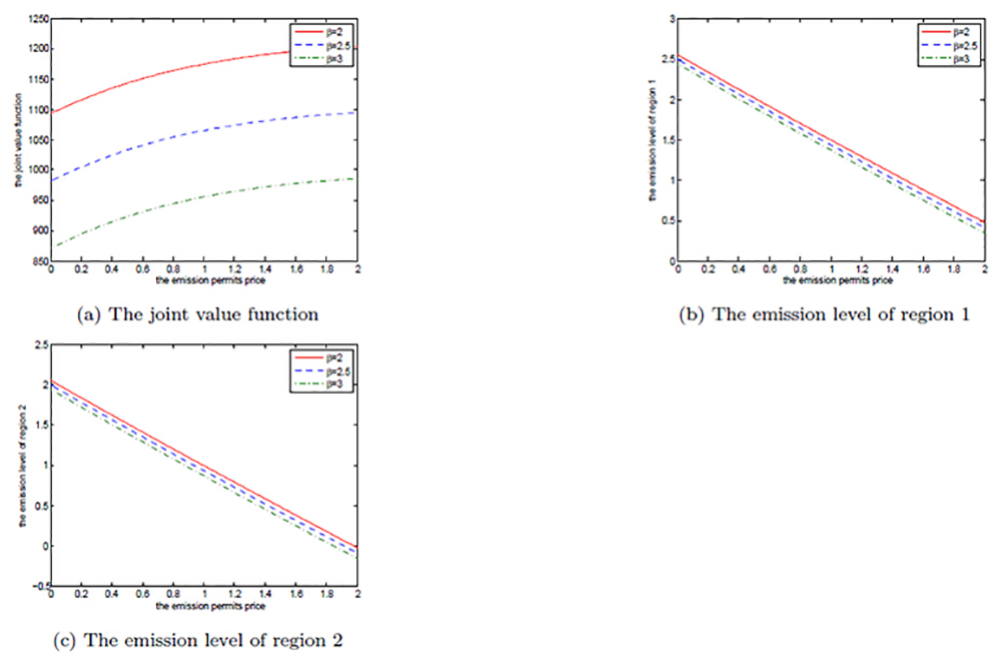
The effects of *β* on the cooperative game.

**Fig 7:**
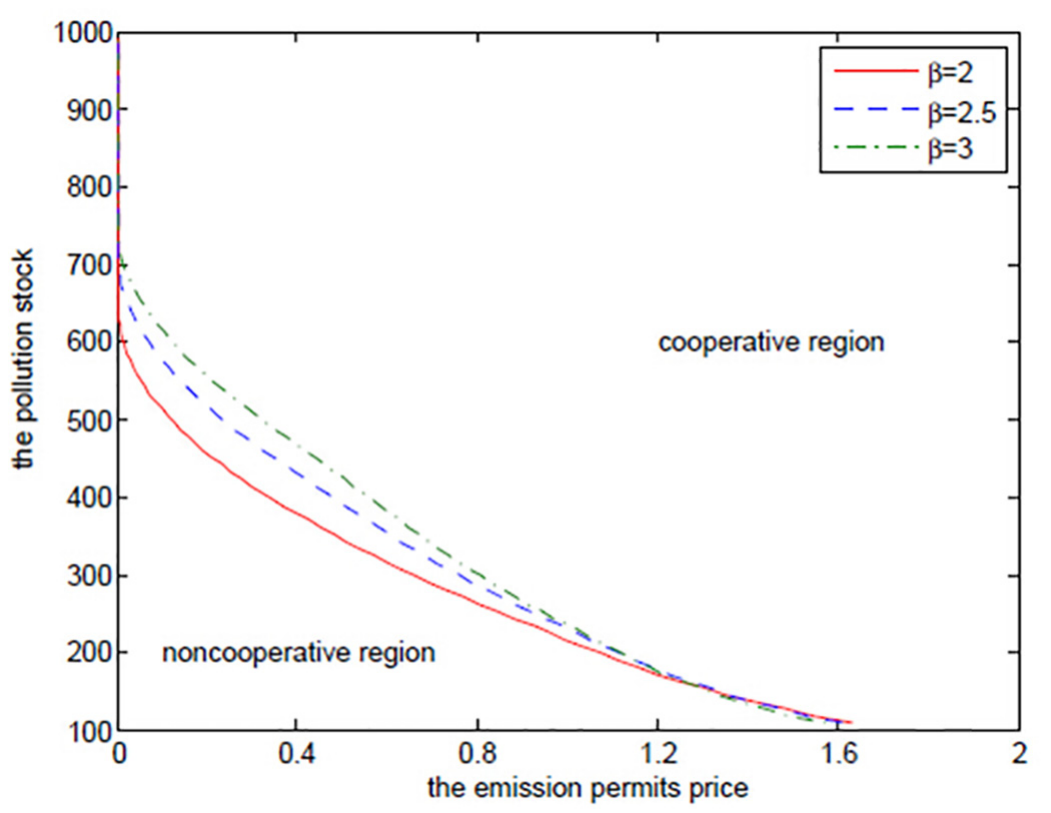
The effects of *β* on the optimal decision boundary.

Analogously, the effects of *β* are illustrated in Figs 5, 6, and 7. In each figure, it is set to be 2, 2.5 and 3, respectively. The parameter *D* in our model measures the suffered pollution damage, and similarly *β* >1 implies that region 2 is more vulnerable than region 1.

In the noncooperative game, a larger *β* means that region 2’s ability in bearing the pollution damage is weaker. Although region 2 reduces its emission level to avoid suffering more pollution damages, its net revenue should also decrease by a wide margin with the increasing *β* due to the reduction in productive revenues. Additionally, region 1 should benefit from the reduced emission, as it can lower the pollution stock without any other change.

In the cooperative game, the two regions make an alliance and should suffer the pollution damage together, so both of them lower their emission levels when *β* increases. However, like the noncooperative case, the joint net revenue should also decrease substantially because of the reduced productive revenues.

Note that we do not use *α* = 0.95 to examine the effects of *β* and the other following parameters on the optimal decision boundary, as in this case the cooperation is always a better decision, and thus there is no optimal decision boundary when *α* = 0.95. For the purpose of illustrating the results clearly, we let *α* equal to other numbers when examining the effects of parameters on the optimal decision boundary in the following discussions.

Fig 7 shows the optimal decision boundaries for different *β*. We can see that the noncooperative region expands with the increasing *β*. Similar to *α*, the farther *β* is from 1, the bigger the difference between the two regions is in the ability of suffering damages, and the smaller the willingness for the advantaged region 1 to cooperate with region 2 is.

Generalizing the emission permits price to be stochastic is our main contribution in this work, so we will examine the effects of the emission permits price parameters: the drift rate *μ*_*S*_ and the volatility *σ*_*S*_ on the value function, the emission level as well as the optimal decision boundary for the two regions.

**Fig 8:**
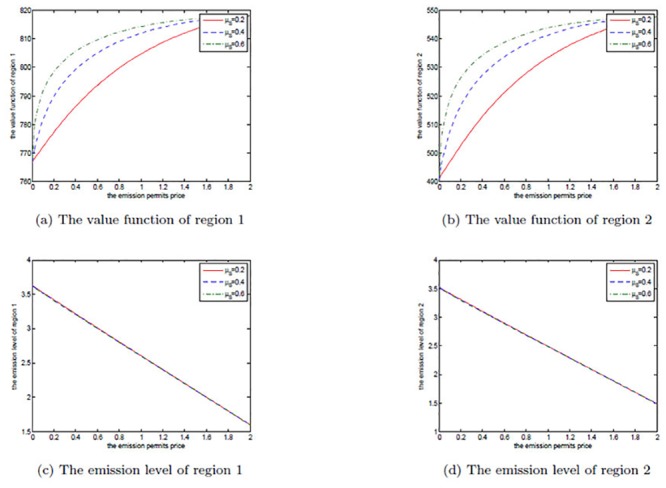
The effects of *μ*_*S*_ on the noncooperative game.

**Fig 9:**
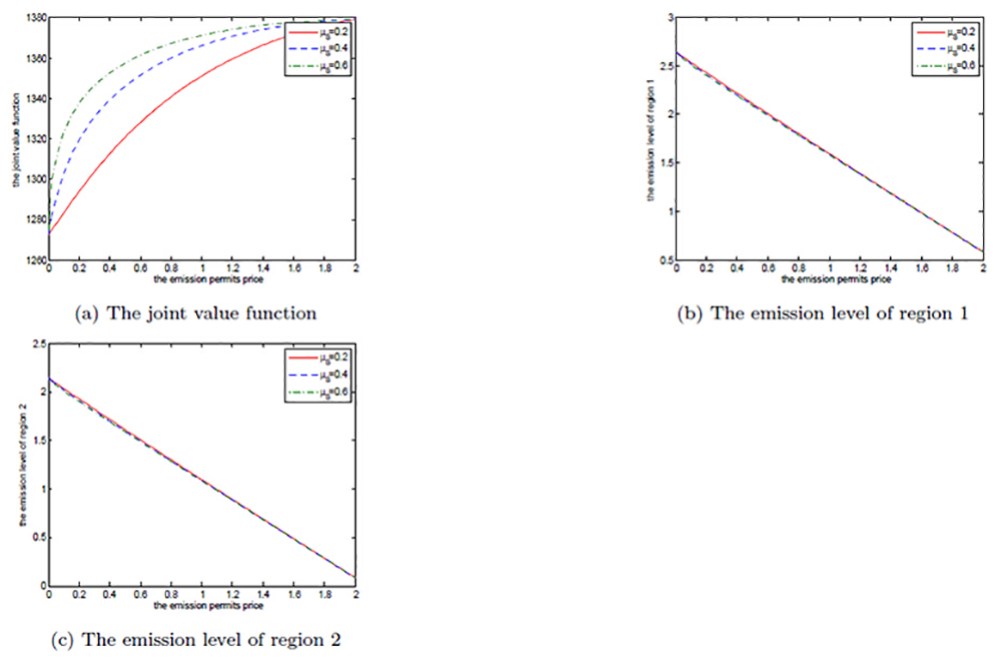
The effects of *μ*_*S*_ on the cooperative game.

**Fig 10:**
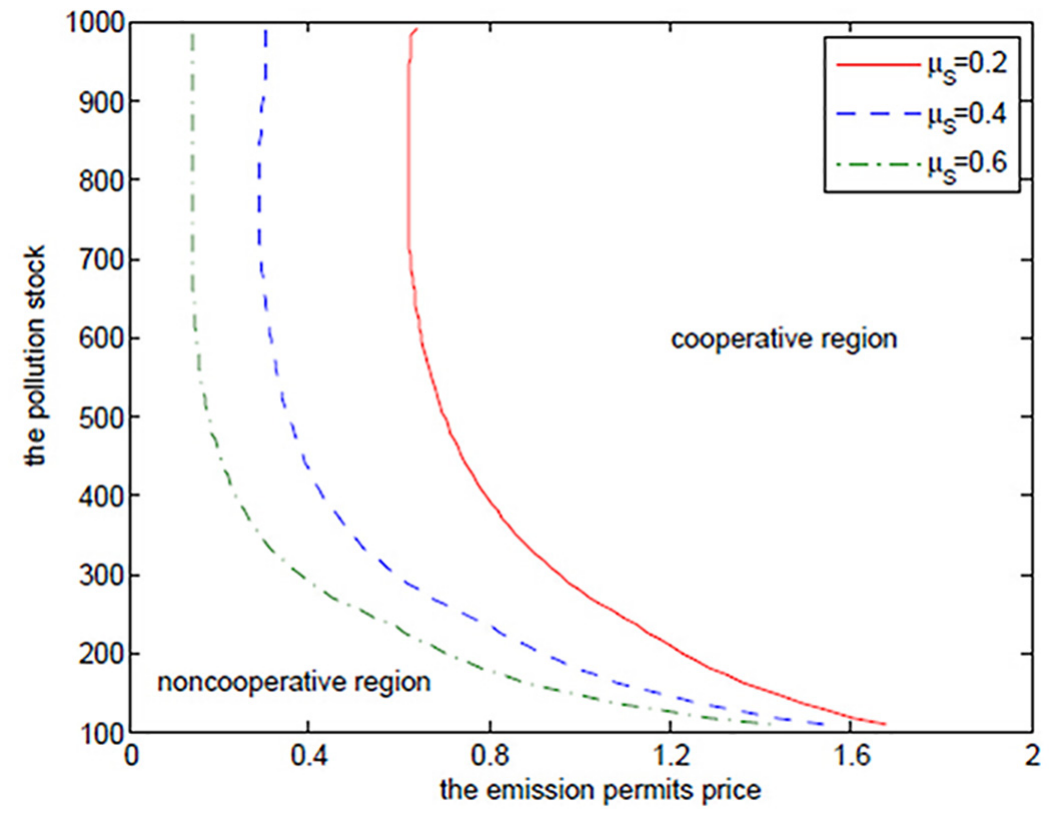
The effects of *μ*_*S*_ on the optimal decision boundary.

The effects of *μ*_*S*_ are shown in Figs 8, 9, and 10. Note that *μ*_*S*_ is set to be 0.2, 0.3, and 0.4 in each figure. We can clearly see that for both the noncooperative and the cooperative games, there is no change in the optimal emission pathes for the two regions. However, the net revenues increase with the increasing *μ*_*S*_. This can be explained as follows.

In Eq (3), the drift rate *μ*_*S*_ is used to model the deterministic trend of the emission permits price, and a larger *μ*_*S*_ means that the price should be also higher. That is to say, with other conditions unchanged both of the two regions can receive more revenues from the emission permits market. So, their net revenues increase with the increasing *μ*_*S*_, while the emission levels do not change in both the noncooperative and the cooperative games.

Obviously, from Fig 10 we can see that the cooperative region expands with the increasing drift rate *μ*_*S*_, in which we fix *α* = 0.6. For the two regions, the emission levels in the cooperative game are lower than the ones in the noncooperative game. As mentioned above, in the cooperation case their emissions are always lower than the initial quota and the unused permits should be sold in the emission permits markets. So, they will prefer to cooperate to save more emission permits and sell them at a higher price to obtain more revenues from the permits markets when the drift rate *μ*_*S*_ is larger.

**Fig 11:**
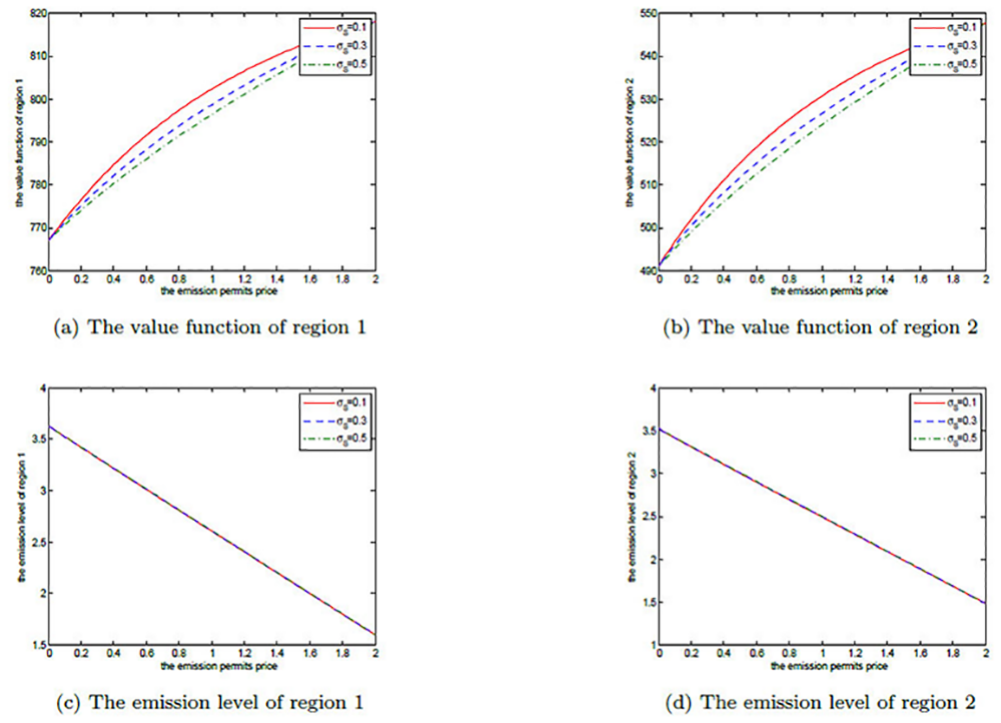
The effects of *σ*_*S*_ on the noncooperative game.

**Fig 12:**
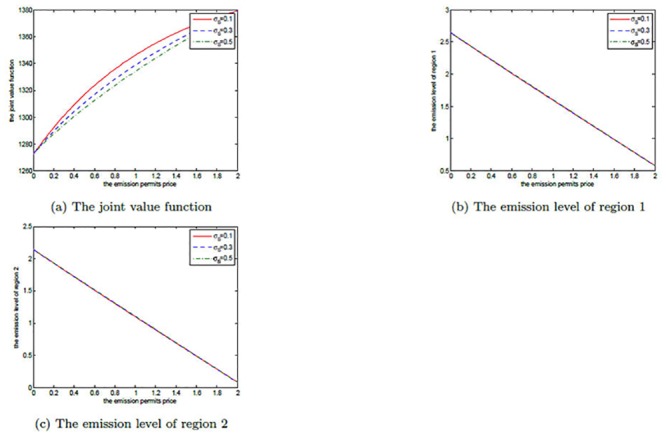
The effects of *σ*_*S*_ on the cooperative game.

**Fig 13:**
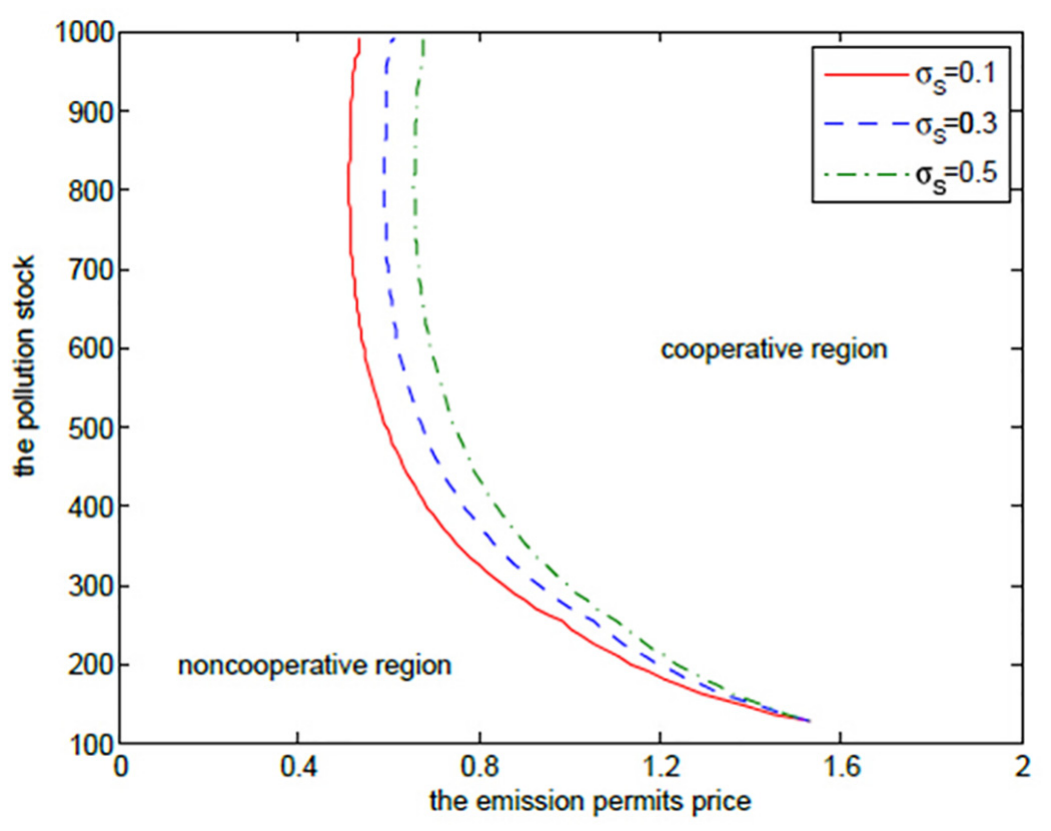
The effects of *σ*_*S*_ on the optimal decision boundary.

Figs 11, 12, and 13 show the effects of *σ*_*S*_ on the noncooperative and the cooperative games, and the optimal decision boundary, respectively. In each figure, *σ*_*S*_ is set to be 0.1, 0.3, and 0.5. Similar to *μ*_*S*_, for both of the noncooperative and the cooperative games, the two regions’ optimal emission paths are not sensitive to *σ*_*S*_. However, the optimal net revenues decrease with the increasing *σ*_*S*_. The reason is that the volatility *σ*_*S*_ measures the uncertainty and the risk of the emission permits price process, and a higher volatility implies that the two players will take on bigger risk on the price. To manage this risk, more efforts, such as investments on strategic portfolios, should be paid. So, a larger volatility of permits price will cut the players’ revenues in the game.

Moreover, the cooperative region in Fig 13 becomes smaller as the volatility *σ*_*S*_ increases. This can be illustrated as follows. The two players want to sell the emission permits saved through their cooperation to earn revenues, however, these revenues may not be realized successfully due to the volatility, and the risk goes up with the increasing volatility. So, the two regions prefer not to cooperate to receive more productive revenues rather than cooperate to seek the risky revenues in permits markets.

#### the effects of parameters *E*_10_ and *E*_20_

**Fig 14:**
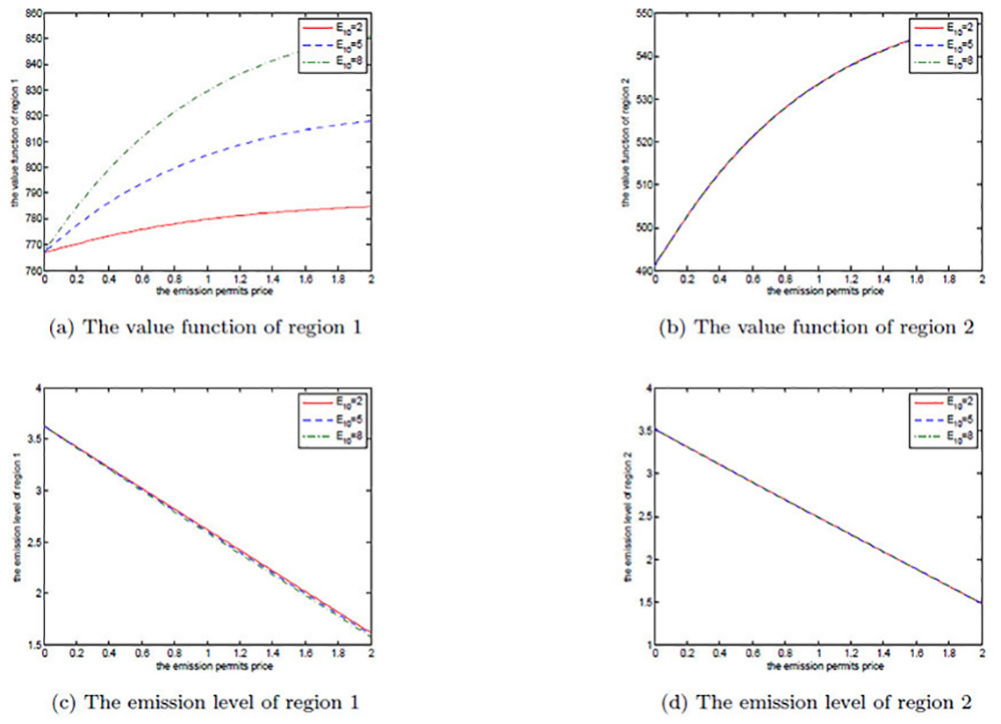
The effects of *E*_10_ on the noncooperative game.

**Fig 15:**
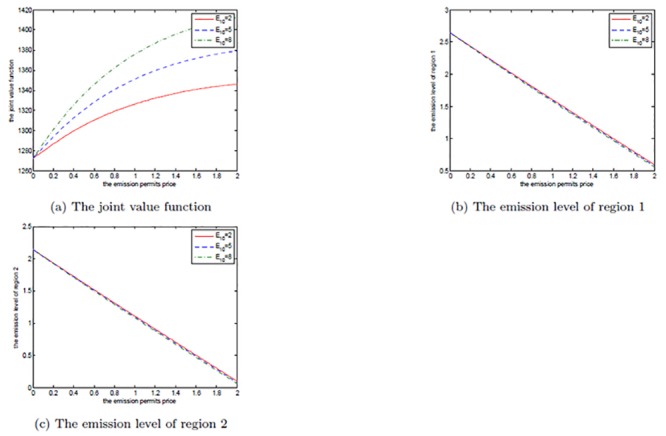
The effects of *E*_10_ on the cooperative game.

**Fig 16:**
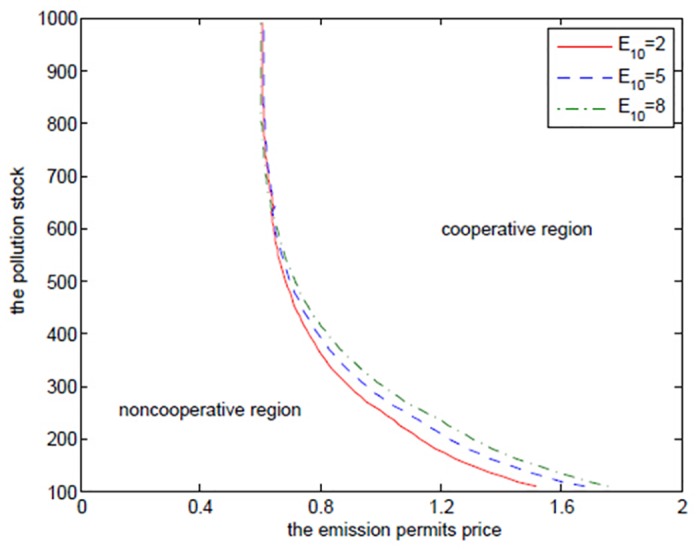
The effects of *E*_10_ on the optimal decision boundary.

**Fig 17:**
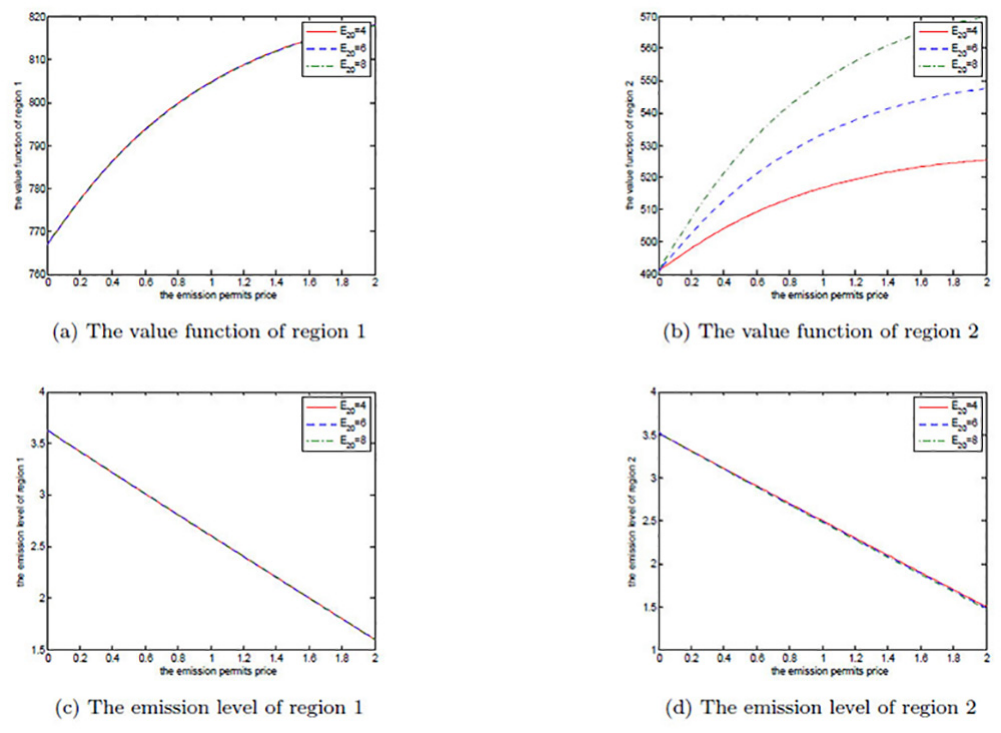
The effects of *E*_20_ on the noncooperative game.

**Fig 18:**
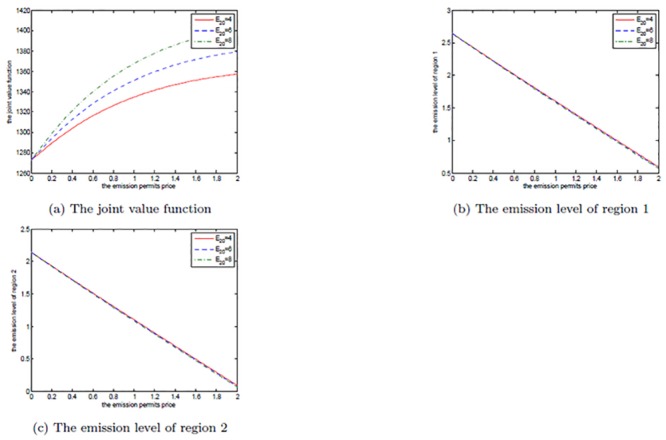
The effects of *E*_20_ on the cooperative game.

**Fig 19:**
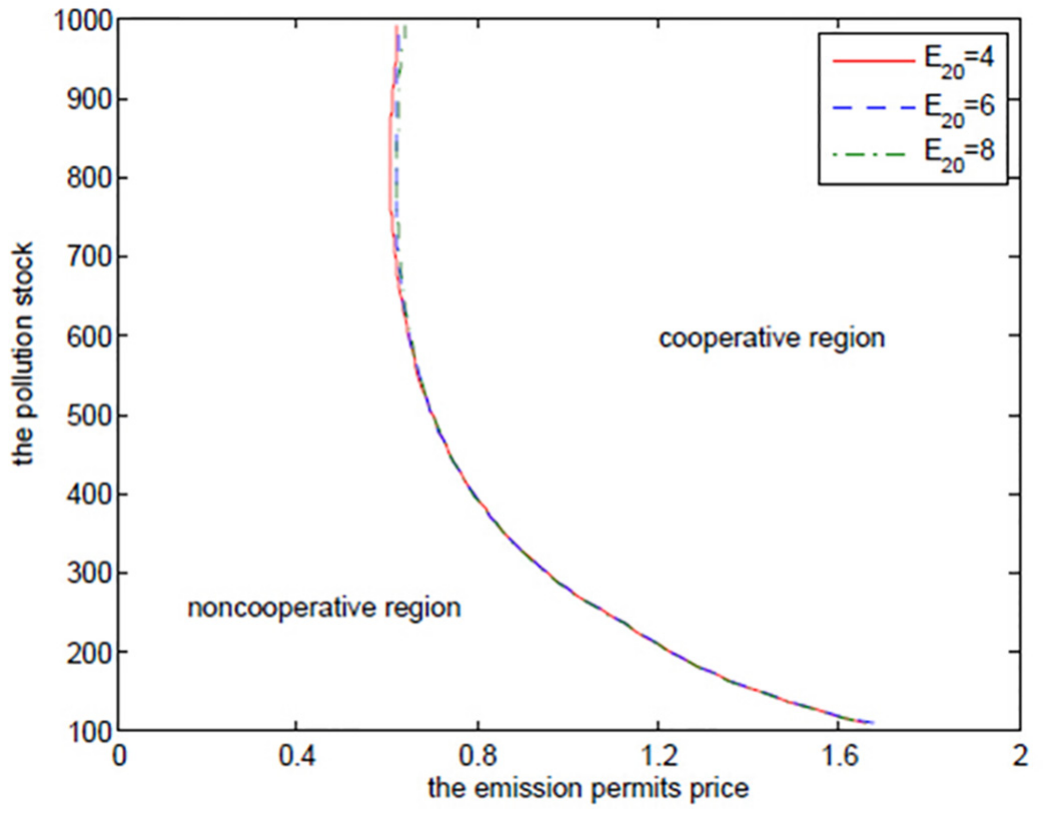
The effects of *E*_20_ on the optimal decision boundary.

The two regions’ initial quotas *E*_10_ and *E*_20_ have also played an important role in this differential game of transboundary industrial pollution with emission permits trading, and they can be regarded as the inherent revenues. The rule of initial quotas’ allocation should be based in part on historical data and emitters’ current actual capabilities. How the initial quotas effect the results are illustrated in Figs 14–19, where *E*_10_ and *E*_20_ are set to be 2, 5, 8 and 4, 6, 8, respectively.

From Figs 14 and 17, we know that the two regions’ initial quotas only influence their own net revenues, respectively, in the noncooperative game and without any changes in emission levels. Moreover, the more the initial quotas are, the greater the net revenues are, which is similar to the cooperative cases presented in Figs 15 and 18. In the cooperative game, the addition in either region’s initial quota can all increase the joint net revenue. Similarly, this is due to the sharing of two regions’ information and resources in the cooperative game.

Figs 16 and 19, where we fix *α* = 0.6 as above, show the effects of two regions’ initial quotas on the optimal decision boundary. We can see from the two figures that the initial quotas, especially the disadvantaged region 2’s, have limited influence on the optimal decision boundary. The reason for the little influence of *E*_10_ is that region 1 may do not want to share the more initial quotas with region 2, and prefers not to cooperate, which is the feature of Fig 16.

## Conclusions and Future Works

In this paper, we present a stochastic differential game of transboundary industrial pollution with the emission permits trading under a finite horizon. More generally, the process of emission permits price is assumed to be stochastic and to follow a GBM. The stochastic optimal control theory has been used by us to derive the HJB equations satisfied by the value functions for the cooperative and the noncooperative games. Then, we propose a so-called fitted finite volume method to solve them. The efficiency and the usefulness of this method are illustrated by the numerical experiments. The two players’ cooperative and noncooperative optimal emission paths, which maximize the regions’ discounted streams of the net revenues, together with the value functions, are obtained. In addition, we can also obtain the threshold conditions for the two regions to decide whether they cooperate or not in different cases. The effects of some parameters on the results have been also examined.

We find that the noncooperation has the potential to be a better decision in the game due to the differences in abilities between the players, and a stochastic emission permits price can realize it. So, we believe that the stochastic price is a more practical and useful tool to be used to model the emission permits trading part of the differential games.

Future works can be performed from two aspects. Mathematically, the convergence rate and the superconvergence of the numerical method can be analysed. The derivation process of the HJB equations should be also improved. From the viewpoint of economics and management, our differential game can be extented to the multi-country or multi-region case for the reason that in practice a region usually has more neighbors than one. Besides, the influence of population growth and technology change on the optimal net revenue and the optimal emission path can be considered.

We anticipate that our methodology from the perspective of partial differential equations combined with numerical methods can make a few contributions to the solving of complex problems in management science.
